# QbD-steered HPTLC approach for concurrent estimation of six co-administered COVID-19 and cardiovascular drugs in different matrices: greenness appraisal

**DOI:** 10.1038/s41598-024-83692-x

**Published:** 2025-02-20

**Authors:** Ahmed R. Mohamed, Rania A. Sayed, Abdalla Shalaby, Hany Ibrahim

**Affiliations:** 1 Pharmaceutical Chemistry Department, Faculty of Pharmacy, Egyptian Russian University, Badr City, Cairo 11829 Egypt; 2 Analytical Chemistry Department, Faculty of Pharmacy, Zagazig University, Zagazig, 44519 Egypt

**Keywords:** COVID-19, Cardiovascular illnesses, HPTLC, Experimental design, Greenness appraisal., Chemistry, Analytical chemistry

## Abstract

Many COVID-19 sufferers have a history of cardiovascular illnesses, which makes them more likely to develop severe COVID-19. Such patients were advised by experts to prioritize drug therapies based on their doctor’s commendations to avoid exacerbating their basic illnesses. Therefore, developing an analytical methodology for the concurrent estimation of medications prescribed for co-treating cardiovascular and COVID-19 illnesses is becoming critical in both bioavailability hubs and QC units. Herein, an inventive, rapid, and affordable HPTLC approach was developed, and its conditions were optimized employing the full factorial design approach for the concurrent estimation of aspirin, atorvastatin, atenolol, losartan, remdesivir, and favipiravir as co-administered medications, either with salicylic acid or not. Using the desirability function, the experimental design approach could forecast the best eluent system for optimal resolution results. On HPTLC-silica plates, the above-mentioned medications were separated utilizing an eluent system of ethyl acetate, methylene chloride, methanol, and ammonia (6:4:4:1 by volume), and their spots were detected at 232 nm. The proposed methodology was evaluated following ICH prerequisites and applied successfully to the medications’ dosage forms, human plasma, and buffered dissolution media with superb recovery proportions and no intrusiveness from formulations’ additives or plasma matrices. Five metrics were employed to appraise the suggested technique’s greenness: AGREE, eco-scale, Raynie and Driver, GAPI, and NEMI. The sensitivity, large sample capacity, and short run duration (15 min) of the proposed methodology confirm its appositeness for regular estimation of the above-mentioned medications.

**Supplementary Information:**

The online version contains supplementary material available at 10.1038/s41598-024-83692-x.

## Introduction

Cardiovascular disorders (CVDs) are one of the most pressing public health concerns as well as the leading cause of death worldwide. Typically, the term “CVDs” refers to a group of illnesses that affect both the heart and blood vessels^1^. Around one-third of all mortalities worldwide in 2016 were attributable to CVDs, and more than 75% of these mortalities took place in nations with low and middle incomes^2,3^. Coronary artery illnesses, thromboembolic disorders, strokes, and hypertension are among the CVDs that cause such mortalities^4^. Fortunately, up to 90% of such illnesses may be conquered by devising or developing diverse remedial strategies that can simultaneously target such illnesses^5^. Many sufferers with CVDs, particularly the elderly, ingest numerous medicines every day, which leads to medication non-compliance. For that reason, fixed-dose combos (polypills) consisting of three or more medications (active constituents) in a single pill were created to enhance ailment control and medication adherence, resulting in a notable decline in disease-related mortality, improved patients’ life quality, and reduced treatment expenditures^6,7^. Among these combos, a mix of aspirin (ASP, an antiplatelet medication), atenolol (ATL, a cardioselective β-blocker medication), atorvastatin calcium (AVC, an antihyperlipidemic medication), and losartan potassium (LSP, an angiotensin-II-receptor blocker medication) was conceived in a fixed-dose pill. This mix was utilized to cure hypertension and lower the danger of both strokes and heart attacks^5^. Salicylic acid (SCA) is ASP’s hydrolytic degradation end product that irritates the stomach when ASP is consumed orally^8^.

COVID-19 is a communicable illness that has swept various nations globally in 2019, killing over 6.20 million people^9,10^. The gravity of this ailment is due to SARS, which influences the lower respiratory tract, triggering fatal pneumonia^11–13^. The likelihood of a grave COVID-19 ailment is greater for people aged 60 or older, women who are pregnant, and individuals with chronic co-morbid conditions like CVD, chronic kidney illness, pulmonary illness, diabetes mellitus, and cancer^9^. Hence, COVID-19 patients who already have basic CVDs should prioritize drug therapies (including ARBs, statins, β-blockers, and also aspirin) as directed by their physician to prevent exacerbation of their basic CVDs, which are closely allied to the higher occurrence of grave COVID-19^14,15^. Despite recent vaccine approvals, this grave global epidemic hasn’t been halted owing to virus mutation, the inadequacy or unavailability of vaccination, and a lack of viable therapeutic replacements^16^. So, in many nations’ protocols, the re-usage of publicly accessible anti-viral medications like remdesivir (RDV) and favipiravir (FPV) is viewed as an efficient and viable option for managing the COVID-19 pandemic^17^. Both FPV and RDV function by obstructing RNA polymerase, which then halts replication of the SARS-CoV-2 virus (the COVID-19 pandemic’s contributory agent)^18,19^. In Table 1, the aforesaid drugs’ chemical structures are exhibited.

**Table 1:**
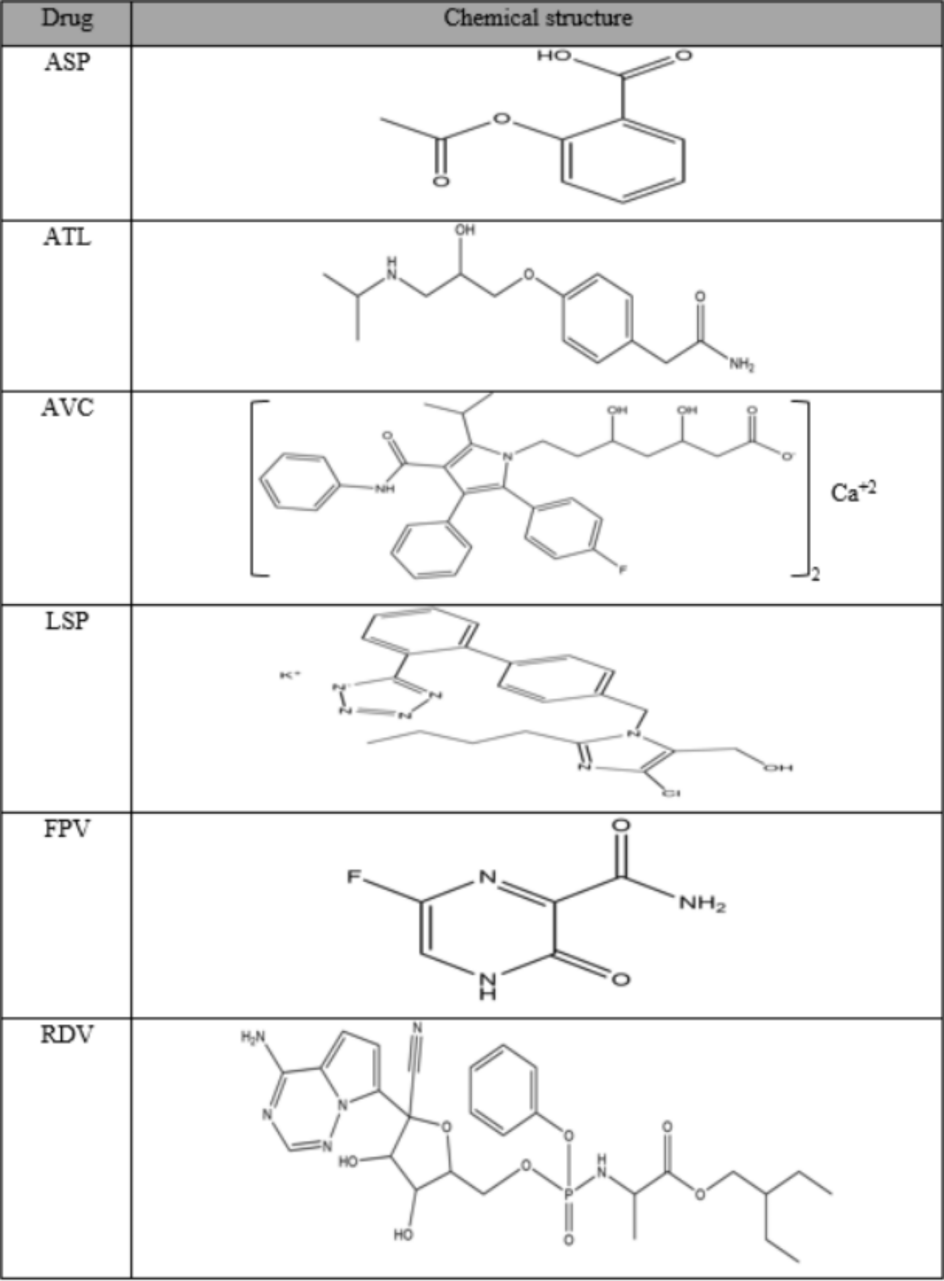
Chemical structure of the six drugs under study.

The available literature disclosed that few RP-HPLC methodologies had been described for concomitant estimation of ATL, ASP, LSP, and AVC, whether in spiked plasma and/or pharmaceutical tablets^20,21^. Whereas, the published methodologies for the concomitant estimation of RDV and FPV were fluorimetry^22^, HPTLC^23^, UHPLC-UV^24^, and UHPLC-MS/MS^25,26^. As of now, the literature doesn’t provide any planar chromatographic techniques explaining the concomitant estimation of the aforesaid six medications. Therefore, the current research presents a delicate, financially viable, selective, and time-saving HPTLC methodology for quick and concomitant estimation of ATL, ASP, LSP, AVC, RDV, and FPV in human plasma and formulations without being hampered by the formulations’ excipients, salicylic acid, or plasma matrices. Moreover, this methodology was utilized to investigate the dissolution patterns of FPV, ATL, ASP, LSP, and AVC in their formulations per the FDA’s suggestions. The suggested HPTLC approach is the first planar technique to date that could separate the aforesaid drugs in a short time and compute their concentrations concurrently in diverse matrices with superb recovery proportions compared to those of the published approaches^21,23^. Because of the short runtime (15 min), the presented HPTLC approach can be leveraged for rapid and low-cost routine assay of the above-mentioned medications in QC labs and/or research centers for bioavailability.

The suggested HPTLC approach was developed and optimized by employing the experimental design approach; full factorial design (FFD) relied on quality by design conceptions which aim to incorporate quality during the development of analytical approaches^27,28^. The experimental design includes an extensive set of statistical designs (models) that assist the analyst in pinpointing the crucial factors influencing the analytical approach’s quality. Moreover, the experimental design helps to investigate the interactive effects of the crucial factors concurrently on the analytical approach’s results and to optimize these factors with minimal experiments to achieve optimal results (optimum resolution, accuracy, and sensitivity for the suggested approach) while saving chemicals and time.

Five appraisal tools, known as AGREE, NEMI, eco-scale (ES), Raynie and Driver (R&D), and GAPI, were presented to appraise the proposed methodology’s greenness for the applied procedures, consumed solvents, and chemicals, revealing a superb greenness report for this methodology relative to the published techniques^21,23^.

## Experimental

### Solvents and materials

All solvents or materials utilized in this work were of analytical grade.

- The ASP standard was a grant from Marcyrl Company (Kaliobeya, Egypt). It received a purity certification of 99.80%.
- Standards of LSP, AVC, ATL, RDV, and FPV were gratuities from EIPICO Company (El Sharkeya, Egypt). They received purity certifications of 99.70%, 99.80%, 99.50%, 99.91%, and 99.68%, respectively.
- Chloroform, ethanol, methanol, hexane, ethyl acetate, methylene chloride, ammonia, salicylic acid, and diethyl ether (Adwic, Egypt).
- Phosphate buffer (5 × 10^− 2^ M aqueous solution, pH = 6.80) (Adwic, Egypt).
- Egypt’s Zagazig University Hospitals supplied drug-free human plasma samples, which were stored at -20 °C till analysis.

### Pharmaceutical preparations.

- Starpill^™^ tablets, made by Cipla Company (India), Lot no. LPH-2207 S, are stated to comprise 50 mg ATL, 50 mg LSP, 10 mg AVC, and 75 mg ASP in a single tablet.
- Avipiravir^®^ tablets, made by EVA Pharma Company (Egypt), Lot no. 2,104,007, are stated to comprise 200 mg FPV in a single tablet.
- Remdesivir-Rameda^®^ vial, made by Rameda Company (Egypt), Lot no. 213,224, is stated to comprise 100 mg RDV in a single vial.

### Apparatus and software

- HPTLC-densitometer consists of a Linomat-5 autosampler (Camag^®^, Hamilton, Switzerland) with a Camag^®^ syringe (22-G, 100-µL) to inject samples under nitrogen stream as spots (bands) on aluminum plates (0.25-mm thickness; 20 × 10 cm) precoated with silica gel 60F_254_ (Darmstadt, Germany). A Camag^®^ glass jar (20 × 10 cm) with a twin trough was employed for mobile phase development. A Camag^®^ TLC scanner 3 S operated with the Wincats^®^ program was employed to scan the HPTLC plates. The instrument was fixed in absorbance scan mode using the deuterium lamp (the instrument’s radiation source). Also, the scanning-light beam’s slit dimensions were set at 3 × 0.45 mm to cover all possible band dimensions (without interloping with the contiguous bands) while the scanning speed was adjusted at 20 mm.s^-1^.
- USP dissolution device (Edition VK 7000, Paddle) was utilized for in-vitro dissolution examinations.
- Sonicator (Edition WUC-A06H), centrifuge (Edition K241R), vortex mixer (Edition VM-300), and Jenway pH meter (Edition 3510) were further used in this work.
- Design expert^®^ 11 software was employed to establish the experimental design exploited to optimize the suggested approach’s chromatographic conditions.

### Standard solutions

Working standard solutions (1 mg.mL^-1^) of the investigated medications (the six drugs) were groomed separately into six 100-mL calibrated flasks by sonicating 100 mg powder of each standard (pure) medication for three minutes in 60 mL methanol. The solutions were then totaled to the mark (100-mL) by methanol.

## Procedures

### Chromatographic conditions

As a stationary phase, HPTLC plates (0.25-mm thickness; 20 × 10 cm) coated with silica gel 60F_254_ were employed for chromatographic separation of the six drugs under study. While the mobile phase (eluent) system was elected by employing the FFD approach, whose model was built by conducting 32 trials on a system of four solvents (ethyl acetate, methylene chloride, methanol, and ammonia) elected after numerous preparatory experiments (Table S1). The designed model included five factors (four solvents and saturation time) and six responses (retardation factors, the six drugs’ R_f_ values). Afterward, the designed model’s factors (five factors) were analyzed and optimized based on pre-established targets (responses) of separation. Consequently, the eluent system was groomed by mingling ethyl acetate-methylene chloride-methanol-ammonia (6:4:4:1 by volume) in the TLC glass jar. This jar was saturated by the eluent vapors for 15 min before placing HPTLC plates inside.

On HPTLC plates, the six drugs’ respective solutions were applied by the µL syringe as discrete compact bands (spots) spaced 15 mm from the HPTLC plates’ bottom edge and 14 mm from one another, with a 3 mm band length. The HPTLC plates were dried after the injection of drugs at ambient temperature (in the open air) and then developed via ascending chromatography in the saturated HPTLC jar up to 8 cm (the migration distance) from the injection (spotting) line. The HPTLC plates were taken out of the glass jar after development and air-dried for a few minutes. Finally, the spots were scanned using the instrument scanner at 232 nm in the absorption scan mode for densitometric analysis of the six drugs.

### Calibration curves construction

By using the Linomat-5 autosampler syringe, different µL aliquots from the specific standard solution (1 mg.mL^-1^) of each medication were injected as bands onto the HPTLC plates (the stationary phase). For every concentration, the injected medications were analyzed three times, as previously stated in Section “Chromatographic conditions”. The final concentrations varied from (0.50–12 µg/band) for ASP, (0.50–15 µg/band) for ATL, (0.10–6 µg/band) for AVC, (0.50–15 µg/band) for LSP, (0.10–5 µg/band) for FPV, and (0.20–5 µg/band) for RDV. At last, the six medications’ calibration charts were established by graphing the peak areas against the corresponding concentrations in µg.band^-1^ and proceeded by calculating the corresponding regression data.

### Applications

#### Pure medications (lab-made mixes)

Precisely measured µL volumes from the respective standard solution of each drug were retracted using the Linomat-5 autosampler syringe and then injected (in triplicate) as bands onto the HPTLC plate. Each band comprised varying proportions of the investigated medications within their linearities. Then, the HPTLC plate was treated, as previously stated in Section “Chromatographic conditions”. At last, the six medications’ concentrations were estimated from their corresponding regression data (regression equations).

#### Pharmaceutical formulations

Five tablets of each Avipiravir^®^ and Starpill™ were weighed independently, crushed finely, and blended homogeneously. Precisely weighed portions of the crushed tablets, equivalent to a single tablet, were relocated independently into two calibrated flasks of 50 mL. Using the sonicator and 10 mL of methanol, the active constituents were twice extracted from the tablet’s excipients. The two developing solutions were then filtrated separately into two additional calibrated flasks of 50 mL utilizing syringe filters of 0.45 μm. The residuals were washed utilizing methanol five times. The calibrated flasks were then totaled with the same solvent to the marks. For the Remdesivir-Rameda^®^ drug vial, a quantity equal to twenty milligrams of RDV’s lyophilized powder was relocated in a calibrated flask (50-mL) holding 30 mL methanol and then sonicated for five minutes. The calibrated flask was then totaled with the same solvent to the mark. After that, appropriate µL aliquots within the investigated drugs’ linearities were retracted from their particular pharmaceutical (tablets or vial) solutions and injected (in triplicate) separately onto the HPTLC plate. The HPTLC plate was treated, as previously stated in Section “Chromatographic conditions”. Eventually, the investigated medications’ nominal quantities in their pharmaceuticals as well as their pure concentrations in µg/band (after implementing the standard addition protocol) were estimated from their corresponding regression data.

#### Spiked plasma

In six groups of 10-mL centrifugation tubes, plasma samples (1 mL) defrosted at the surrounding temperature were spiked with diverse standard aliquots of the six drugs. The tubes were then vortexed for sixty seconds (to denaturize the proteins) after adding three mL aliquots of methanol^29^. At 5000 rpm, the tubes’ insides were centrifuged for fifteen minutes to segregate the proteins in full. All tubes’ clear supernatants were prudently segregated and dried completely at the surrounding temperature. Following drying, the residues were gently reconstituted with two mL aliquots of methanol before being filtered straight into six groups of 10-mL calibrated flasks via 0.45-µm syringe filters. All filtrates were subsequently diluted to 10 mL with methanol. Blank (drug-free) samples were similarly and concurrently processed. Different µL aliquots of the groomed samples were injected (in triplicate) onto the HPTLC plates to attain final concentrations varying from (1–10) µg/band for each ASP, ATL, and LSP while ranging from (0.50–5) µg/band for AVC and (0.50–3) µg/band for each FPV and RDV. The HPTLC plates were treated, as previously stated in Section “Chromatographic conditions”. At last, the six medications’ calibration charts and regression data were generated to estimate their groomed concentrations (in µg/band) in plasma.

#### Dissolution testing

As per FDA’s regulations^30^, the USP device-II (Paddle) was utilized to test the dissolution of ATL, LSP, AVC, and ASP from their tablets (Starpill^™^ tablets) at 37.00 ± 0.50 °C with a 75 RPM stirring speed. The dissolution medium comprised 0.50 L of phosphate buffer (50 mM, pH 6.80). Initially, five mL aliquots were retracted from each dissolution vessel at 5-minute to 60-minute intervals, filtrated via 0.45-µm syringe filters, and injected (in triplicate) onto the HPTLC plate. The retracted volumes were superseded at every time interval with a new 5 mL dissolution medium. Lastly, the HPTLC plate was treated, as previously stated in Section “Chromatographic conditions”, to estimate the investigated medications’ release proportions and to create their plots (profiles) of dissolution. Similarly, the FPV dissolution from its tablets (Avipiravir^®^ tablets) was performed, but at 3-minute to 60-minute intervals.

## Results and discussion

The HPTLC techniques have a number of merits, like large sample capacity, quick analysis, high reproducibility, sharp peaks, minimal solvent use, high-resolution power, and low detection thresholds, along with their applications in biomedical and pharmaceutical analysis^23,31^. Therefore, the current research presents a validated affordable HPTLC technique for quick and concomitant analysis of ATL, ASP, LSP, AVC, RDV, and FPV in diverse matrices (pure forms, human plasma, dosage forms, and dissolution medium) with little environmental impact.

### Method development and optimization

The procedure (chromatographic conditions) of the suggested approach was optimized to attain accurate results, good resolution, and symmetric sharp peaks with acceptable R_f_ values as follows.

#### Eluent composition

The crucial step in developing the HPTLC approach is identifying the best eluent system. So, a number of experiments were conducted employing various eluent systems with variable compositions and ratios. The tested systems included ethyl acetate: methanol: ammonia (10:4:0.50 by volume), ethyl acetate: hexane: methanol: ammonia (10:1:1:0.20 by volume), ethyl acetate: methanol: chloroform: ammonia (10:2:1:0.20, 9:4:0.50:0.50, 10:4:0.50:0.50, 10:3:1:0.50, 10:4:1:0.50, and 3:1:6:0.50 by volume), chloroform: diethyl ether: methanol: ammonia (5:3:2:0.50 by volume), ethyl acetate: methylene chloride: ethanol: ammonia (6:2:2:1 by volume), and lastly ethyl acetate: methylene chloride: methanol: ammonia (8:2:5:0.75, 9:1:5:0.50, 10:1:4:0.50, 10:1:4:0.75, 10:1.50:4:0.75, 7:3:5:0.50, 6:2:2:1, 6:2:3:0.50, 6:3:4:0.20, and 6:3:4:0.75 by volume). The eluent systems were investigated for saturation times of 10 and 15 min.

**Fig. 1:**
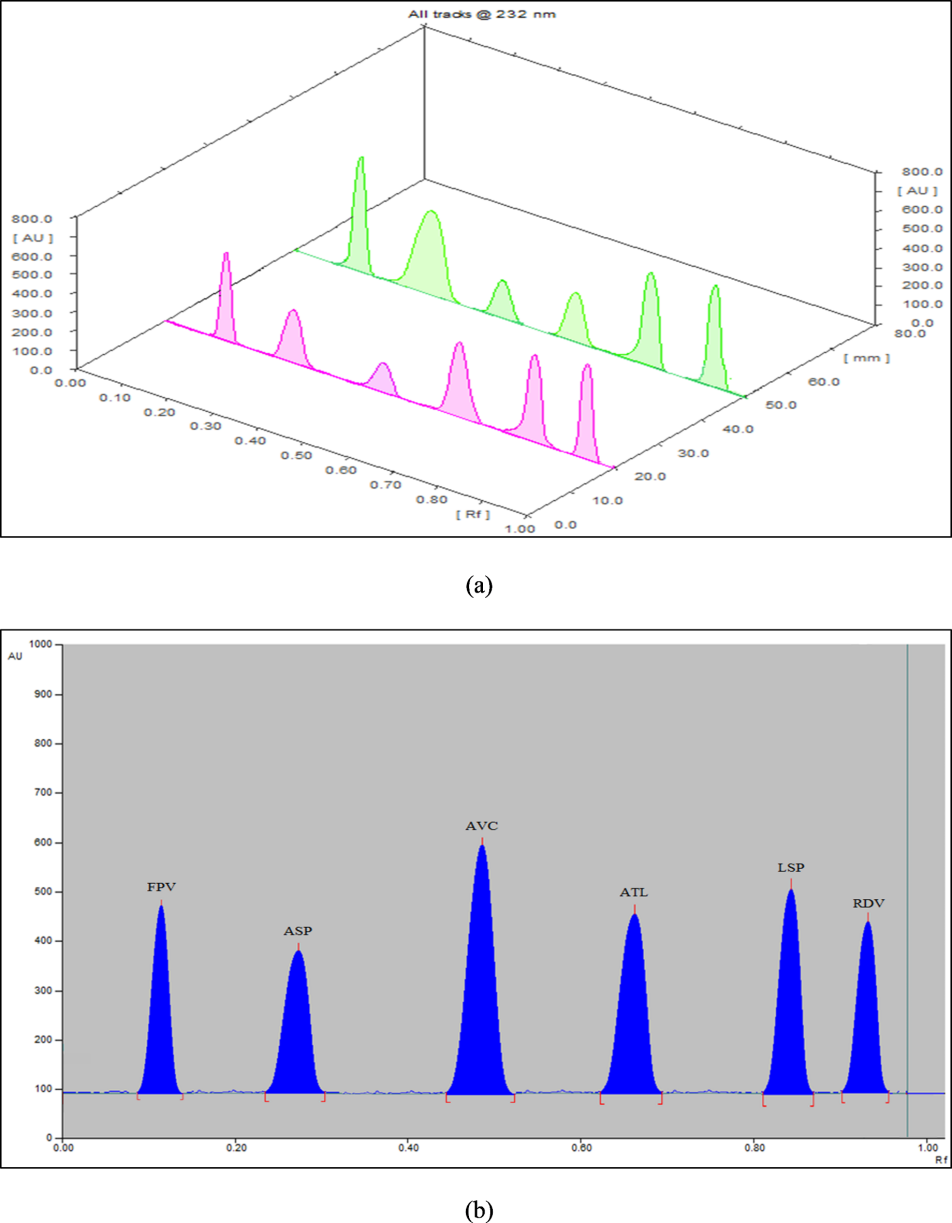
(**a**) Three-dimensional and (**b**) Two-dimensional HPTLC-densitograms of separated peaks of FPV (R_f_ = 0.12), ASP (R_f_ = 0.27), AVC (R_f_ = 0.49), ATL (R_f_ = 0.67), LSP (R_f_ = 0.84), and RDV (R_f_ = 0.95) under the specified chromatographic conditions.

**Fig. 2:**
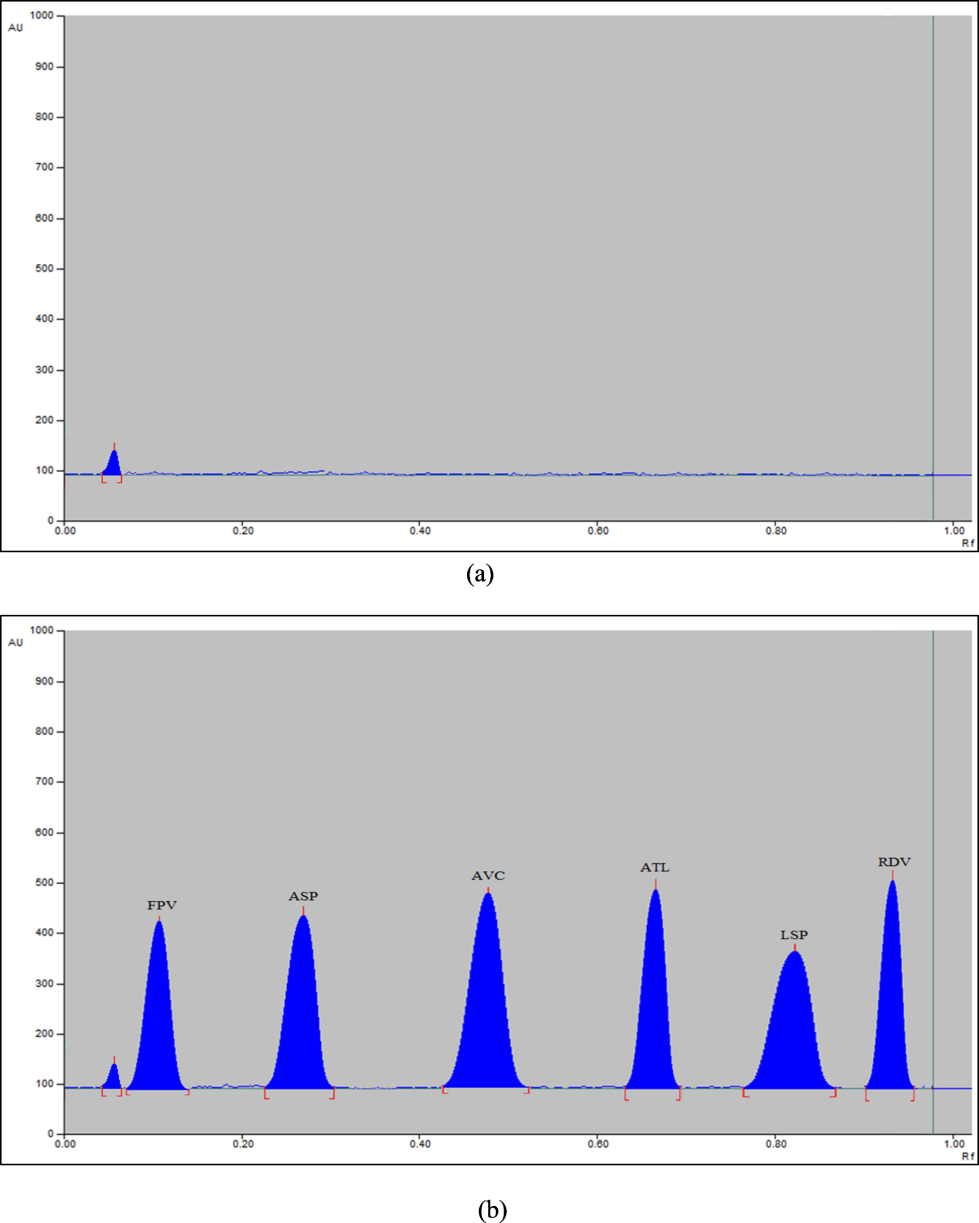
(**a**) HPTLC-densitogram of blank plasma sample. (**b**) HPTLC-densitogram of plasma sample spiked with FPV (1.50 µg/band), ASP (5 µg/band), AVC (3 µg/band), ATL (5 µg/band), LSP (5 µg/band), and RDV (1.50 µg/band).

The mixture of ethyl acetate, methylene chloride, methanol, and ammonia (6:3:4:0.75 by volume) was the only eluent system that offered satisfactory separation without peak tailing. For best resolution results, the aforesaid eluent system (composition and saturation time) was optimized using the FFD approach. The statistical model developed from the FFD approach forecasted that the mixture of ethyl acetate, methylene chloride, methanol, and ammonia (6:4:4:1 by volume) at 15 min (saturation time) would produce optimal separation results. With sharp peaks, the optimized eluent system resulted in R_f_ values of 0.12, 0.27, 0.49, 0.67, 0.84, and 0.95 for FPV, ASP, AVC, ATL, LSP, and RDV, respectively (Fig. 1). The optimized eluent system was apt for human plasma applications (Fig. 2) as well as for separating salicylic acid (Fig. S1).

Indeed, the conventional optimization of any analytical approach depends on altering one variable (univariate procedures) while maintaining the other variables constant. Accordingly, it doesn’t investigate the interactive effects between the experimental variables concurrently. In contrast, optimization through experimental design approaches (e.g. FFD approach) permits studying the influence of all variables concurrently and investigating their interactive effects on the response(s). So, the FFD approach was adopted to optimize the suggested approach’s conditions for better resolution results.

To build the FFD approach’s statistical model, the crucial factors (variables) affecting the six drugs’ elution results and the responses should be identified. The chosen eluent system for further optimization was ethyl acetate: methylene chloride: methanol: ammonia (6:3:4:0.75 by volume). From the chosen system’s earlier trials, the crucial variables were saturation time and volumes of ethyl acetate, methylene chloride, methanol, and ammonia while the responses were the six drugs’ R_f_ values. The designed model included 32 trials performed to investigate the influence of each crucial variable and their interactive effects on the six drugs’ R_f_ values (Table S1).

Afterward, the designed model was analyzed statistically by ANOVA for each studied drug to verify the model’s significance. For all six drugs, the designed model was statistically significant (because P-values were < 0.05) and showed high R^2^ (correlation coefficient) values varying from (0.95 to 0.98) and high predicted R^2^ values in reasonable accord with values of adjusted R^2^ (because their differences were < 0.20), indicating the model’s high predictability and acceptable fitting. The ANOVA results and fit statistics of the designed model were summarized in Tables S2 and S3, in order. Additionally, the designed model was evaluated for each studied drug using the normal plots of residuals, which exhibited a straight line, denoting the normal spread of residuals and the agreement between the observed (actual) values of R_f_ and predicted ones by the model (Fig. S2). Other plots, called residuals versus predicted plots, exhibited random scatter of the predicted R_f_ values and residuals, denoting the constant variation among data (Fig. S3). Also, the residuals versus run plots (Fig. S4) showed a random distribution of residuals without outliers within each trial (run), denoting the accuracy of the designed model. The residuals (or externally studentized residuals) presented in Figs. S2-S4 were employed to identify any analysis-related problems.

For optimal results, the crucial variables were optimized simultaneously based on pre-established targets of separation using the desirability function (its values range from zero to one). The targets were set numerically as R_f_ values at 0.12, 0.26, 0.48, 0.66, 0.84, and 0.94 for FPV, ASP, AVC, ATL, LSP, and RDV, in order. Out of 100 solutions found for the pre-established R_f_ values, the designed model revealed one solution (eluent system), with the best desirability of 0.96. The solution ramps (Fig. S5), overlay plot (Fig. S6), and 3D-surface plots (Fig. S7) exhibited that the eluent system of ethyl acetate, methylene chloride, methanol, and ammonia (6:4:4:1 by volume) at 15 min (saturation time) could achieve the desirability of 0.96. The perturbation plots displayed in Fig. S8 were created to elucidate the influence of each crucial variable on the R_f_ value. Finally, five confirmatory trials were conducted to assess the suggested solution (eluent system) by the model, as listed in Table S4, where the observed and predicted R_f_ values were very close.

#### Scanning wavelength

Densitometric measurements were implemented at diverse wavelengths (220, 232, 240, and 250 nm). For all six medications, the wavelength of 232 nm exhibited satisfactory sensitivity with untailed peaks and minimum noise (Fig. 1).

#### Band dimensions

The six drugs were applied onto the HPTLC plates as bands. Therefore, various band dimensions were trialed to get symmetrical, sharp, and well-resolved peaks. The ideal bandwidth was elected to be 0.30 cm to avert bands from diffusion outside scanning tracks during elution (mobile phase development). As well, the bands’ interspaces were elected to be 1.40 cm to keep the contiguous bands from intruding.

## Method validation

The following ICH guides^32^ were pursued to appraise the proposed HPTLC methodology.

### Linearity

Pursuant to the ideal circumstances, the suggested methodology’s linearity (linear range) was assessed by gauging the densitometric peak areas (responses) at 232 nm in triplicate for diverse concentrations of the investigated medications. Hence, the calibration charts were generated by graphing the densitometric peak areas against the investigated medications’ concentrations in µg.band^− 1^. In Table 2, the linearities and correlation coefficients, besides other regression data, were registered, signifying superb linear ranges.

**Table 2:** Assay parameters for the determination of the six drugs by the suggested HPTLC approach.

Drugs	ASP	ATL	AVC	LSP	FPV	RDV
Parameters						
Linearity range (µg/band)	0.50–12	0.50–15	0.10–6	0.50–15	0.10–5	0.20–5
Correlation coefficient (r)	0.9999					
Slope	513.46	353.09	560.36	372.45	770.97	807.99
Intercept	-5.77	-3.72	4.15	-27.86	0.69	6.49
S.D of intercept ^a^	21.40	14.31	3.48	11.37	3.61	13.95
LOD (µg/band)	0.14	0.13	0.02	0.10	0.01	0.05
LOQ (µg/band) ± SD ^b^	0.42 ± 0.03	0.40 ± 0.02	0.06 ± 0.01	0.30 ± 0.04	0.05 ± 0.01	0.17 ± 0.02

^a^ Standard deviation of intercept.

^b^ Mean of three determinations.

### LOD and LOQ

The LODs’ and LOQs’ values were estimated in µg.band^− 1^ to evaluate the proposed methodology’s delicacy. In Table 2, the aforesaid values were registered for all investigated medications, signifying the proposed methodology’s high delicacy.

### Accuracy and precision

The accuracy as well as the two precision types (repeatability and intermediate precision) were valued by scrutinizing (in triplicate) three pure concentrations casing the investigated medications’ linearities employing the proposed methodology. The scrutiny was conducted for repeatability on one day and for intermediate precision on three chronological days. The accuracy findings were registered as recovery% ± SD in Table S5, where all precision findings were also registered as RSD% (< 2%). The aforesaid findings, besides the Er%, which didn’t outstrip ± 2%, were acceptable, demonstrating the proposed methodology’s high levels of precision, reliability, and accuracy.

### Specificity

The proposed methodology’s specificity was valued by scrutinizing five lab-made mixes involving diverse proportions of the investigated medications within their linear ranges (Table S6). The findings in Table S6, besides the chromatograms (2D and 3D) presented in Fig. 1 and S1, divulge that the proposed methodology was successful in separating as well as estimating the investigated medications without any intermeddling with each other and/or with salicylic acid. The specificity was also valued by applying the proposed methodology for quantifying the investigated medications in their formulations, generating decent recoveries without any meddling from the formulations’ additives and/or salicylic acid.

### Robustness

The proposed methodology’s robustness was valued by scrutinizing the sway of small intended disparities in ideal circumstances on the proposed methodology’s analytical functioning. The intended disparities were in the solvent system proportion (ethyl acetate, methylene chloride, methanol, and ammonia contents ± 5%), saturation time (± 3 min.), developing distance (± 0.30 cm), and detection wavelength (± 2 nm). Lastly, the small disparities in the above-mentioned factors divulged trifling variances in recovery% compared to the epitome circumstances, demonstrating the proposed methodology’s robustness and reliability (Table S7).

### System suitability

To guarantee that the solvent system was functioning appropriately, system suitability factors were scrutinized in relation to retardation factors, capacity factors, resolutions, selectivity factors, and tailing factors. The estimated factors in Table S8 were all inside the permissible or reference thresholds^33^, implying the proposed methodology’s appositeness for separating as well as estimating the investigated medications under the epitome circumstances.

## Method applications

### Pharmaceutical formulations

The investigated medications were successfully estimated in their particular formulations by utilizing the proposed methodology (Table 3). The listed outcomes in the aforesaid table were reasonable and closely matched the formulations’ labeled quantities without intermeddling with the formulations’ additives, demonstrating the proposed methodology’s appositeness for regular estimation of the investigated medications in QC units. The proposed methodology’s validity was scrutinized by implementing the standard addition protocol, generating decent recoveries (Table 3).

**Table 3:** Determination of the six drugs by the suggested HPTLC approach in their pharmaceuticals and application of standard addition approach.

Product		Product (Found% ± SD)*	Standard addition (Pure found% ± SD)*
Starpill™ tablets	ASP	98.31 ± 0.39	100.49 ± 0.99
	ATL	99.80 ± 0.69	100.18 ± 0.68
	AVC	100.98 ± 0.32	100.05 ± 1.54
	LSP	99.66 ± 1.08	99.82 ± 0.84
Avipiravir^®^ tablets	FPV	100.93 ± 0.38	100.49 ± 1.19
Remdesivir-Rameda^®^ vial	RDV	100.11 ± 0.24	99.94 ± 1.02

*Mean of five determinations.

### Spiked plasma

The proposed methodology’s high delicacy (in terms of small LOD values) was utilized for estimating the investigated medications in human plasma samples (Table 4). The outcomes in the preceding table and Fig. 2’s chromatograms divulge that the proposed methodology can be applied to studies on the bioequivalence of the investigated medications without intermeddling with plasma matrices.

**Table 4:** Validation parameters for quantification of the six drugs by the suggested HPTLC approach in spiked human plasma.

Drugs	ASP	ATL	AVC	LSP	FPV	RDV
Parameters						
Concentration range (µg/band)	1–10	1–10	0.50–5	1–10	0.50–3	0.50–3
Correlation coefficient (r)	0.9997	0.9998				
Slope	443.73	338.82	226.92	347.24	751.48	782.80
Intercept	8.38	3.20	185.62	-12.89	-10.60	-15.06
S.D of intercept	34.02	22.87	6.40	23.99	16.10	15.26
LOD (µg/band)	0.25	0.22	0.09	0.23	0.07	0.06
LOQ (µg/band) ± SD ^a^	0.76 ± 0.05	0.67 ± 0.03	0.28 ± 0.02	0.69 ± 0.04	0.21 ± 0.02	0.19 ± 0.01
Accuracy ^b^ (Mean ± SD)	100.76 ± 0.94	99.98 ± 1.18	100.30 ± 0.93	100.02 ± 0.93	99.89 ± 1.32	101.02 ± 1.40
Intra-day precision (RSD%)	0.65	0.43	0.72	0.85	0.97	0.35
Inter-day precision (RSD%)	1.12	1.62	1.19	1.14	1.75	1.58

^a^ Mean of three determinations.

^b^ Mean of five determinations.

### Dissolution testing

Dissolution testing (DT) is an imperative procedure for the quality control of pharmaceuticals and for foretelling the precise timing of active constituents’ dose delivery to the patient^34,35^. Hence, DT establishes a link between in-vivo and in-vitro patterns. In this work, DT was applied to both Avipiravir^®^ and Starpill™ tablets to examine the investigated medications’ release from these pharmaceuticals (tablets). The investigated medications’ release proportions were estimated using the proposed methodology and then charted in contrast to time intervals to create these medications’ dissolution profiles (Fig. S9).

## Greenness appraisal

Concerning drug synthesis or analysis, the vast majority of labs internationally are now transitioning to green chemistry. This is accomplished by restraining the utilization of cancer-causing solvents or swapping them with greener ones in order to diminish their detrimental sways on both analysts and the environment. Hence, five appraisal tools, known as ES, GAPI, NEMI, R&D, and AGREE, were presented to get an extensive description concerning the proposed and published^21,23^ methodologies’ greenness profiles.

Concerning the ES tool^36^, all penalty points as well as ES scores for both proposed and published methodologies were estimated (Table S9), illuminating the proposed methodology’s supremacy from a greenness standpoint.

Concerning the GAPI tool, a fifteen-piece pictogram (circular shape) was conceived, casing diverse analytical paces from sampling (sample collection) to final sample detection and estimation^31^. Three colored ciphers were utilized to shadow each piece: red, yellow, or green, which, respectively, emblematize low, medium, or high ecological peculiarities. The proposed and published methodologies’ GAPI profiles were conceived in Table S10, exposing the proposed methodology’s supremacy from a greenness standpoint.

Concerning the NEMI tool, the proposed and published methodologies’ greenness was valued by utilizing a four-piece circular pictogram emblematic of the methodologies’ perilousness, corrosivity, toxicity, and quantity of trash spawned^37^. Each piece was granted a white or green cipher, implying either non-green or green traits. The NEMI profiles were conceived in Table S10 for both proposed and published methodologies, illuminating the proposed methodology’s supremacy from a greenness standpoint.

Concerning the R&D tool, the proposed and published methodologies’ greenness was valued by utilizing a pictogram (pentagon shape) of a five-triangle symbolizing five classes: environmental hazard, energy, waste amount, safety hazard, and health hazard^38^. Each triangle was granted a color: red, yellow, or green, implying low, medium, or high obedience to the criteria for green analytical chemistry (GAC), respectively. The R&D profiles for both proposed and published methodologies were conceived in Table S10, illuminating the proposed methodology’s supremacy from a greenness standpoint.

Concerning the AGREE tool, a twelve-piece pictogram (circular shape) was conceived, casing all GAC criteria by utilizing AGREE’s open-source program^39^. Each piece’s color ranged from dark red to dark green as per environmental sway, while the appraisal score was estimated at the AGREE pictogram’s midpoint. The AGREE profiles for both proposed and published methodologies with total appraisal scores were conceived in Table S10, illuminating the proposed methodology’s supremacy from a greenness standpoint.

As per the aforementioned greenness appraisal tools, we deduce that the proposed HPTLC methodology is greener than the published^21,23^ methodologies, disclosing its appropriateness for routinely determining the investigated medications in diverse matrices, either in QC workrooms or in bioavailability hubs with little environmental sway, at the most affordable price.

## Statistical analysis

The outcomes accomplished by the proposed methodology were compared statistically to those accomplished by the published^21,23^ methodologies for the investigated medications’ analysis in their pure forms. As well, the F-test values (for precision) and t-test ones (for accuracy) were estimated for all medications without overriding the referential thresholds (Table S11). Concerning Table S11’s statistical analysis, there aren’t perceptible variances in functioning between the proposed methodology and the published methodologies, implying the proposed methodology’s appositeness for routinely determining the investigated medications in QC centers.

## Conclusion

An eclectic, precise, and robust HPTLC methodology was created for the first time, and its conditions were optimized employing the FFD approach for quick and concurrent estimation of ATL, ASP, LSP, AVC, RDV, and FPV as co-administered medications, irrespective of SCA presence. The proposed methodology could estimate the above-mentioned medications in diverse matrices with superb recovery proportions without being hampered by plasma matrices or formulations’ additives. The virtues of low detection thresholds, quick analysis, and large sample capacity render the proposed methodology apt for routinely determining the above-mentioned medications, either in bioavailability hubs or in QC centers, at the most affordable price. The proposed methodology’s greenness was valued by utilizing five appraisal tools, which exposed its supremacy over the published methodologies in this regard.

## Electronic supplementary material

Below is the link to the electronic supplementary material.

Supplementary Material 1

## Data Availability

All data generated or analysed during this study are included in this published article [and its supplementary information files].
