# Supplementary material for: QbD-steered HPTLC approach for concurrent estimation of six co-administered COVID-19 and cardiovascular drugs in different matrices: greenness appraisal
Source: Sci Rep. 2025 Feb 20;15:6252. doi: 10.1038/s41598-024-83692-x (PMC11842590; doi:10.1038/s41598-024-83692-x)
Supplement: Supplementary file 1 — Supplementary Material 1 [file 41598_2024_83692_MOESM1_ESM.docx]

**Table S1: Two-level (2^5^) full factorial design and responses.**

| Design order | | Factor  1 | Factor  2 | Factor  3 | Factor  4 | Factor  5 | Response 1 | Response 2 | Response 3 | Response 4 | Response 5 | Response 6 |
| --- | --- | --- | --- | --- | --- | --- | --- | --- | --- | --- | --- | --- |
| Std | Run | Ethyl acetate (mL) | Methylene chloride (mL) | Methanol  (mL) | Ammonia  (mL) | Saturation time (min) | FPV (R_f_) | ASP (R_f_) | AVC (R_f_) | ATL (R_f_) | LSP (R_f_) | RDV (R_f_) |
| 16 | 1 | 7 | 4 | 5 | 1 | 10 | 0.10 | 0.22 | 0.44 | 0.61 | 0.79 | 0.89 |
| 1 | 2 | 6 | 3 | 4 | 0.75 | 10 | 0.10 | 0.19 | 0.34 | 0.57 | 0.62 | 0.75 |
| 28 | 3 | 7 | 4 | 4 | 1 | 15 | 0.10 | 0.26 | 0.48 | 0.65 | 0.85 | 0.91 |
| 14 | 4 | 7 | 3 | 5 | 1 | 10 | 0.11 | 0.21 | 0.41 | 0.59 | 0.76 | 0.87 |
| 6 | 5 | 7 | 3 | 5 | 0.75 | 10 | 0.10 | 0.20 | 0.31 | 0.52 | 0.61 | 0.72 |
| 10 | 6 | 7 | 3 | 4 | 1 | 10 | 0.10 | 0.22 | 0.43 | 0.60 | 0.78 | 0.88 |
| 18 | 7 | 7 | 3 | 4 | 0.75 | 15 | 0.10 | 0.20 | 0.32 | 0.54 | 0.61 | 0.74 |
| 2 | 8 | 7 | 3 | 4 | 0.75 | 10 | 0.11 | 0.21 | 0.40 | 0.51 | 0.62 | 0.74 |
| 15 | 9 | 6 | 4 | 5 | 1 | 10 | 0.11 | 0.29 | 0.43 | 0.59 | 0.81 | 0.91 |
| 9 | 10 | 6 | 3 | 4 | 1 | 10 | 0.11 | 0.27 | 0.48 | 0.66 | 0.81 | 0.91 |
| 31 | 11 | 6 | 4 | 5 | 1 | 15 | 0.11 | 0.29 | 0.50 | 0.65 | 0.87 | 0.94 |
| 12 | 12 | 7 | 4 | 4 | 1 | 10 | 0.10 | 0.26 | 0.49 | 0.64 | 0.84 | 0.90 |
| 22 | 13 | 7 | 3 | 5 | 0.75 | 15 | 0.10 | 0.20 | 0.30 | 0.51 | 0.61 | 0.71 |
| 3 | 14 | 6 | 4 | 4 | 0.75 | 10 | 0.10 | 0.19 | 0.34 | 0.56 | 0.63 | 0.75 |
| 21 | 15 | 6 | 3 | 5 | 0.75 | 15 | 0.11 | 0.18 | 0.34 | 0.54 | 0.62 | 0.76 |
| 25 | 16 | 6 | 3 | 4 | 1 | 15 | 0.11 | 0.27 | 0.47 | 0.67 | 0.81 | 0.91 |
| 30 | 17 | 7 | 3 | 5 | 1 | 15 | 0.11 | 0.22 | 0.41 | 0.60 | 0.76 | 0.87 |
| 5 | 18 | 6 | 3 | 5 | 0.75 | 10 | 0.11 | 0.18 | 0.34 | 0.55 | 0.61 | 0.76 |
| 26 | 19 | 7 | 3 | 4 | 1 | 15 | 0.10 | 0.22 | 0.41 | 0.59 | 0.77 | 0.88 |
| 19 | 20 | 6 | 4 | 4 | 0.75 | 15 | 0.10 | 0.20 | 0.34 | 0.55 | 0.63 | 0.75 |
| 4 | 21 | 7 | 4 | 4 | 0.75 | 10 | 0.10 | 0.20 | 0.29 | 0.50 | 0.61 | 0.72 |
| 7 | 22 | 6 | 4 | 5 | 0.75 | 10 | 0.11 | 0.20 | 0.32 | 0.51 | 0.61 | 0.73 |
| 29 | 23 | 6 | 3 | 5 | 1 | 15 | 0.11 | 0.21 | 0.43 | 0.60 | 0.85 | 0.92 |
| 24 | 24 | 7 | 4 | 5 | 0.75 | 15 | 0.10 | 0.21 | 0.31 | 0.53 | 0.62 | 0.72 |
| 8 | 25 | 7 | 4 | 5 | 0.75 | 10 | 0.10 | 0.21 | 0.31 | 0.53 | 0.61 | 0.72 |
| 17 | 26 | 6 | 3 | 4 | 0.75 | 15 | 0.10 | 0.19 | 0.33 | 0.58 | 0.63 | 0.75 |
| 23 | 27 | 6 | 4 | 5 | 0.75 | 15 | 0.11 | 0.21 | 0.31 | 0.51 | 0.60 | 0.73 |
| 32 | 28 | 7 | 4 | 5 | 1 | 15 | 0.10 | 0.22 | 0.43 | 0.62 | 0.80 | 0.89 |
| 20 | 29 | 7 | 4 | 4 | 0.75 | 15 | 0.10 | 0.21 | 0.29 | 0.51 | 0.62 | 0.72 |
| 13 | 30 | 6 | 3 | 5 | 1 | 10 | 0.11 | 0.21 | 0.42 | 0.60 | 0.85 | 0.92 |
| 27 | 31 | 6 | 4 | 4 | 1 | 15 | 0.12 | 0.27 | 0.50 | 0.67 | 0.86 | 0.95 |
| 11 | 32 | 6 | 4 | 4 | 1 | 10 | 0.11 | 0.24 | 0.45 | 0.62 | 0.82 | 0.91 |

**Table S2: ANOVA results for the designed factorial model for the six drugs.**

| Drugs  Parameters | ASP | ATL | AVC | LSP | FPV | RDV |
| --- | --- | --- | --- | --- | --- | --- |
| Sum of squares | 0.03 | 0.08 | 0.14 | 0.32 | 0.001 | 0.23 |
| Degree of freedom | 10 | 8 | 8 | 6 | 17 | 5 |
| Mean square | 0.003 | 0.01 | 0.02 | 0.05 | 0.0001 | 0.05 |
| F-value | 41.33 | 57.81 | 70.30 | 149.08 | 15.65 | 424.51 |
| p-value | < 0.0001 (significant model) | | | | | |

**Table S3: Fit statistics for the designed factorial model for the six drugs.**

| Drugs  Parameters | ASP | ATL | AVC | LSP | FPV | RDV | Ideal value |
| --- | --- | --- | --- | --- | --- | --- | --- |
| Model R^2^ | 0.95 | 0.95 | 0.96 | 0.97 | 0.95 | 0.99 | 1 |
| Adjusted R^2^ | 0.93 | 0.94 | 0.95 | 0.97 | 0.89 | 0.98 | 1 |
| Predicted R^2^ | 0.89 | 0.91 | 0.92 | 0.95 | 0.74 | 0.98 | 1 |
| Adequate Precision | 21.81 | 24.15 | 22.47 | 29.34 | 14.99 | 48.95 | > 4 |
| Standard deviation | 0.008 | 0.01 | 0.02 | 0.02 | 0.002 | 0.01 | < 2 |
| Mean | 0.22 | 0.58 | 0.39 | 0.71 | 0.11 | 0.82 | - |
| Coefficient of variation% | 3.77 | 2.32 | 4.15 | 2.67 | 1.80 | 1.28 | < 10% |

**Table S4: Confirmatory trials to investigate the validity of the suggested solution.**

| **Response**  **(R_f_)*** | **Predicted mean** | **Predicted median** | **Observed mean** | **Standard deviation** | **Standard error predicted** | **95% PI low** | **95% PI high** |
| --- | --- | --- | --- | --- | --- | --- | --- |
| FPV | 0.12 | 0.12 | 0.12 | 0.002 | 0.002 | 0.11 | 0.12 |
| ASP | 0.26 | 0.26 | 0.26 | 0.008 | 0.006 | 0.24 | 0.27 |
| AVC | 0.48 | 0.48 | 0.49 | 0.02 | 0.01 | 0.46 | 0.50 |
| ATL | 0.65 | 0.65 | 0.66 | 0.01 | 0.009 | 0.63 | 0.67 |
| LSP | 0.84 | 0.84 | 0.84 | 0.02 | 0.01 | 0.81 | 0.86 |
| RDV | 0.93 | 0.93 | 0.93 | 0.01 | 0.006 | 0.92 | 0.94 |

*Mean of five determinations.

**Table S5: Accuracy and precision data obtained for the determination of the six drugs by the suggested HPTLC approach.**

| Drugs  Parameters | ASP | ATL | AVC | LSP | FPV | RDV |
| --- | --- | --- | --- | --- | --- | --- |
| Accuracy |  | | | | | |
| Mean ± SD | 99.42 ± 0.82 | 100.01 ± 1.07 | 98.55 ± 0.52 | 100.66 ± 0.37 | 100.65 ± 0.52 | 98.72 ± 0.48 |
| RSD% | 0.82 | 1.07 | 0.53 | 0.37 | 0.52 | 0.49 |
| Er%^a^ | -0.58 | 0.01 | -1.45 | 0.66 | 0.65 | -1.28 |
| Intra-day precision^b^ |  | | | | | |
| Mean ± SD | 99.93 ± 0.56 | 100.91 ± 0.36 | 100.80 ± 0.16 | 99.44 ± 0.74 | 101.32 ± 0.70 | 98.70 ± 0.61 |
| RSD% | 0.56 | 0.36 | 0.16 | 0.74 | 0.69 | 0.62 |
| Er%^a^ | -0.07 | 0.91 | 0.80 | -0.56 | 1.32 | -1.30 |
| Inter-day precision^c^ |  | | | | | |
| Mean ± SD | 99.31 ± 0.74 | 99.78 ± 1.13 | 99.09 ± 0.91 | 100.48 ± 0.94 | 99.92 ± 1.44 | 100.04 ± 1.53 |
| RSD% | 0.75 | 1.13 | 0.92 | 0.94 | 1.44 | 1.53 |
| Er%^a^ | -0.69 | -0.22 | -0.91 | 0.48 | -0.08 | 0.04 |

^a^ Relative error percentage.

^b^ The intra-day analysis, average of three concentrations (2, 6, and 10 μg/band) for each ASP, ATL, and LSP while (0.50, 2, and 4 μg/band) for each AVC, FPV, and RDV, repeated three times within one day.

^c^ The inter-day analysis, average of three concentrations (2, 6, and 10 μg/band) for each ASP, ATL, and LSP while (0.50, 2, and 4 μg/band) for each AVC, FPV, and RDV, repeated three times within three sequential days.

**Table S6: Determination of the six drugs in their laboratory-made mixtures by the suggested HPTLC approach.**

| Mix | ASP | | ATL | | AVC | | LSP | | FPV | | RDV | |
| --- | --- | --- | --- | --- | --- | --- | --- | --- | --- | --- | --- | --- |
|  | Added^a^ | R%^b^ | Added^a^ | R%^b^ | Added^a^ | R%^b^ | Added^a^ | R%^b^ | Added^a^ | R%^b^ | Added^a^ | R%^b^ |
| Mix 1 | 4 | 99.18 | 4 | 99.25 | 2 | 98.08 | 2 | 100.30 | 2 | 100.34 | 2 | 98.30 |
| Mix 2 | 3 | 98.62 | 3 | 98.61 | 3 | 99.41 | 3 | 100.84 | 3 | 100.40 | 3 | 99.70 |
| Mix 3 | 1 | 99.42 | 3 | 99.86 | 1 | 100.60 | 3 | 101.80 | 1 | 99.25 | 3 | 100.21 |
| Mix 4 | 4 | 100.16 | 2 | 99.42 | 4 | 100.97 | 2 | 99.87 | 4 | 99.90 | 2 | 100.22 |
| Mix 5^c^ | 7.50 | 101.62 | 5 | 100.67 | 1 | 101.22 | 5 | 101.34 | 1 | 100.15 | 1 | 101.04 |
| Mean  ±  SD | 99.80  ±  1.16 | | 99.56  ±  0.76 | | 100.05  ±  1.30 | | 100.83  ±  0.77 | | 100.008  ±  0.46 | | 99.89  ±  1.01 | |

^a^ Added (μg/band).

^b^ Recovery% (mean of three determinations).

^c^ The same ratio of ASP, ATL, AVC, and LSP in Starpill^TM^ tablets.

**Table S7: Robustness study of the suggested HPTLC approach using concentration (4 μg/band) for each studied drug.**

| Variation | | | ASP  (4 μg/band) | ATL  (4 μg/band) | AVC  (4 μg/band) | LSP  (4 μg/band) | FPV  (4 μg/band) | RDV  (4 μg/band) |
| --- | --- | --- | --- | --- | --- | --- | --- | --- |
|  |  |  | Recovery%^a^ ± SD | | | | | |
| No variation  (optimal conditions^b^) | | | 100.34 ± 1.08 | 99.90 ± 1.10 | 99.66 ± 0.57 | 99.50 ± 0.53 | 100.33 ± 0.82 | 101.46 ± 0.72 |
| Mobile phase composition | Ethyl acetate | +5% | 100.02 ± 0.39 | 99.96 ± 1.29 | 99.16 ± 0.78 | 99.98 ± 0.77 | 100.34 ± 1.04 | 99.92 ± 1.39 |
|  |  | −5% | 100.40 ± 1.01 | 99.61 ± 0.84 | 99.61 ± 0.41 | 100.40 ± 1.43 | 100.18 ± 0.50 | 100.79 ± 1.10 |
|  | Methylene chloride | +5% | 99.81 ± 1.58 | 99.82 ± 1.04 | 101.95 ± 0.70 | 100.21 ± 1.05 | 99.60 ± 1.33 | 98.67 ± 0.60 |
|  |  | −5% | 100.36 ± 1.09 | 99.32 ± 1.24 | 101.38 ± 0.98 | 99.87 ± 0.63 | 100.19 ± 0.40 | 98.73 ± 1.33 |
|  | Methanol | +5% | 100.59 ± 0.92 | 99.70 ± 0.82 | 101.52 ± 0.37 | 99.41 ± 1.11 | 99.97 ± 0.80 | 98.98 ± 1.18 |
|  |  | −5% | 100.85 ± 0.67 | 99.89 ± 0.68 | 101.65 ± 0.67 | 99.83 ± 0.41 | 100.27 ± 0.34 | 99.33 ± 0.83 |
|  | Ammonia | +5% | 100.09 ± 1.51 | 99.34 ± 0.29 | 101.05 ± 1.03 | 99.44 ± 0.77 | 99.97 ± 0.86 | 99.03 ± 1.02 |
|  |  | −5% | 99.58 ± 0.85 | 98.81 ± 0.67 | 100.38 ± 0.60 | 99.81 ± 0.92 | 100.16 ± 0.52 | 99.39 ± 0.76 |
| Saturation time | +3 min. | | 99.28 ± 0.98 | 98.87 ± 1.21 | 99.90 ± 0.60 | 99.61 ± 1.14 | 99.96 ± 0.58 | 99.21 ± 0.86 |
|  | −3 min. | | 99.88 ± 0.99 | 99.14 ± 1.36 | 100.08 ± 0.51 | 99.49 ± 0.76 | 100.02 ± 0.69 | 99.18 ± 0.82 |
| Developing distance | +0.3 cm | | 99.57 ± 1.23 | 99.50 ± 0.85 | 101.01 ± 1.19 | 99.70 ± 1.34 | 99.39 ± 1.03 | 101.72 ± 1.01 |
|  | −0.3 cm | | 98.91 ± 1.33 | 99.86 ± 1.07 | 101.72 ± 1.20 | 100.35 ± 1.47 | 100.50 ± 1.57 | 100.37 ± 1.55 |
| Detection wavelength | +2 nm | | 99.52 ± 1.16 | 99.71 ± 1.25 | 101.66 ± 1.07 | 100.23 ± 1.25 | 99.86 ± 1.42 | 100.36 ± 1.66 |
|  | −2 nm | | 99.21 ± 1.20 | 99.55 ± 0.70 | 101.38 ± 1.01 | 100.16 ± 1.34 | 99.14 ± 0.94 | 98.99 ± 1.31 |

^a^ Mean of three determinations.

^b^ Optimal conditions: mobile phase composition (ethyl acetate: methylene chloride: methanol: ammonia, 6:4:4:1 by volume), saturation time (15 min.), developing distance (8 cm), and detection wavelength (232 nm).

**Table S8: System suitability parameters of the suggested HPTLC approach for the determination of the six drugs.**

| Parameters | FPV | ASP | AVC | ATL | LSP | RDV | Reference value [33] |
| --- | --- | --- | --- | --- | --- | --- | --- |
| Retardation factor (R_f_) | 0.12 | 0.27 | 0.49 | 0.67 | 0.84 | 0.95 | 0−1 |
| Capacity factor (K′) | 7.33 | 2.70 | 1.04 | 0.49 | 0.19 | 0.05 | 0−10 |
| Resolution (Rs) | 2.45 | 3.01 | 2.38 | 2.57 | 1.93 |  | >1.5 |
| Selectivity factor (α) | 2.71 | 2.59 | 2.12 | 2.58 | 3.80 |  | >1 |
| Tailing factor (T) | 0.90 | 0.88 | 0.96 | 0.86 | 0.91 | 0.86 | T= 1 for a symmetric peak |

**Table S9: Results of eco-scale analysis for determination of the six drugs employing the suggested HPTLC approach.**

| Approaches  Parameters | HPTLC | Reported approach [21] | Reported approach [23] |
| --- | --- | --- | --- |
| **Reagents** |  | | |
| Ethyl acetate | 4 | – | 4 |
| Methylene chloride | 4 | – | – |
| Methanol | 6 | 6 | 6 |
| Ammonia | 6 | – | 6 |
| Ethanol | – | – | 4 |
| Acetonitrile | – | 8 | 4 |
| Orthophosphoric acid | – | 2 | – |
| Dipotassium hydrogen phosphate | – | 0 | – |
| **Instruments**  HPLC/ TLC-densitometer |  | | |
| Energy | 1  [> 0.1 kWh/sample] | 1  [> 0.1 kWh/sample] | 1  [> 0.1 kWh/sample] |
| Occupational hazard | 0 | 3 | 0 |
| Waste | 3 | 5 | 3 |
| Waste treatment | 3 | 3 | 3 |
| Total penalty points | Σ 27 | Σ 28 | Σ 31 |
| Analytical eco-scale total score ^a,b^ | 73 | 72 | 69 |
|  | Acceptable green analysis | Acceptable green analysis | Acceptable green analysis |

^a^ Analytical eco-scale total score = 100–total penalty points.

^b^ If the score is > 75, it indicates excellent green analysis.

If the score is > 50, it indicates acceptable green analysis.

If the score is < 50, it indicates inadequate green analysis.

**Table S10: Greenness assessment of the suggested and reported [21,23]** **approaches by GAPI, NEMI, Raynie and Driver, and AGREE tools.**

| Reported approach [23] | Reported approach [21] | Suggested HPTLC approach | Tool |
| --- | --- | --- | --- |
| 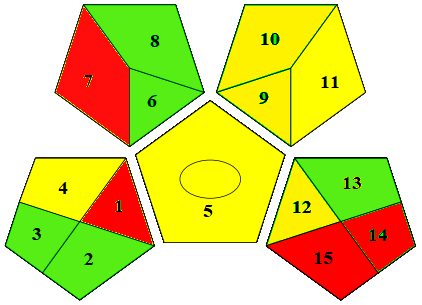 | 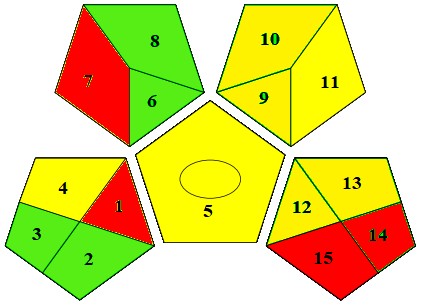 | 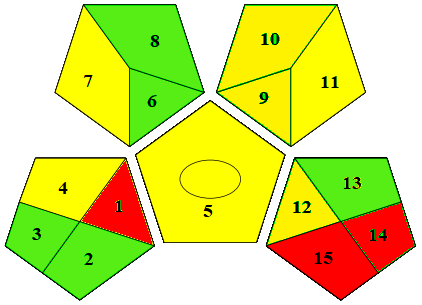 | GAPI |
| 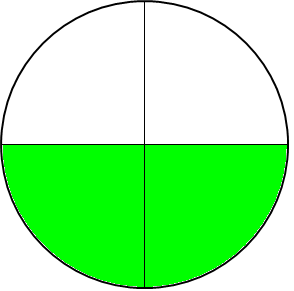 | 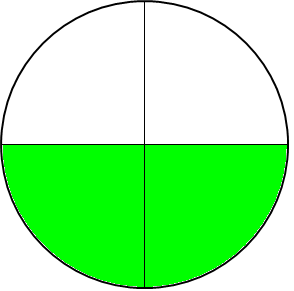 | 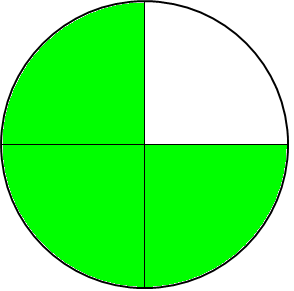 | NEMI |
| 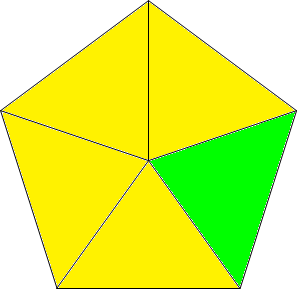 | 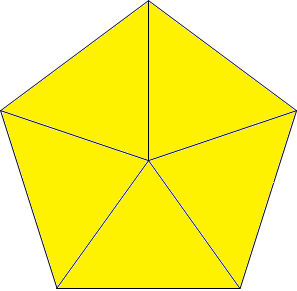 | 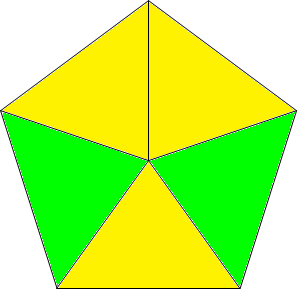 | Raynie and Driver |
| 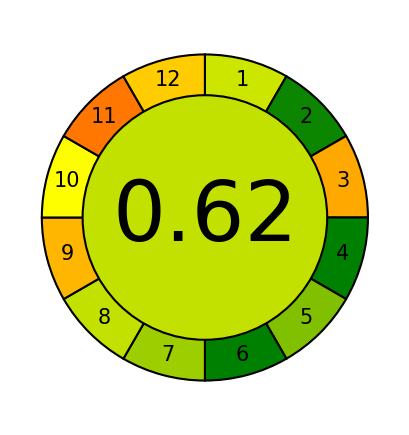 | 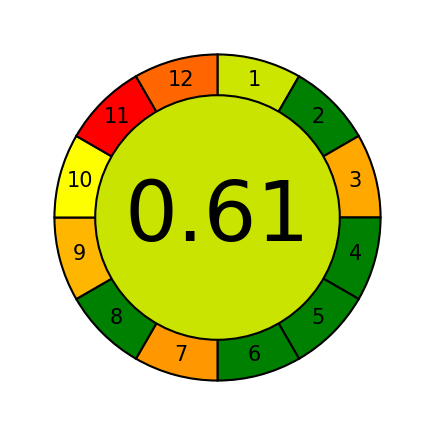 | 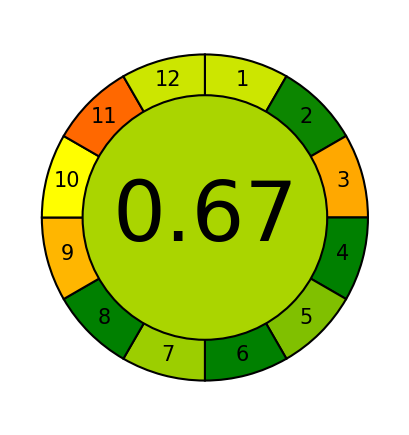 | AGREE |

**Table S11: Statistical comparison between the suggested and reported [21,23]** **approaches for the analysis of the six drugs in their pure forms.**

| Approaches  Parameters | Suggested HPTLC approach | | | | | | Reported approach [21] | | | | Reported approach [23] | |
| --- | --- | --- | --- | --- | --- | --- | --- | --- | --- | --- | --- | --- |
|  | ASP | ATL | AVC | LSP | FPV | RDV | ASP | ATL | AVC | LSP | FPV | RDV |
| Mean | 99.78 | 99.67 | 100.22 | 99.77 | 100.21 | 99.75 | 100.34 | 100.40 | 99.71 | 99.27 | 100.52 | 100.08 |
| SD | 0.56 | 0.97 | 1.17 | 0.49 | 0.73 | 0.99 | 1.05 | 1.27 | 0.91 | 0.30 | 0.97 | 1.02 |
| N | 5 | 5 | 5 | 5 | 5 | 5 | 5 | 5 | 5 | 5 | 5 | 5 |
| Variance | 0.31 | 0.94 | 1.37 | 0.24 | 0.53 | 0.98 | 1.10 | 1.61 | 0.83 | 0.09 | 0.94 | 1.04 |
| Student^'^s  *t*-test (2.31)* | 1.05 | 1.02 | 0.77 | 1.95 | 0.57 | 0.52 | ------- | ------- | ------- | ------- | ------- | ------- |
| F- value (6.39)* | 3.52 | 1.71 | 1.65 | 2.67 | 1.77 | 1.06 | ------- | ------- | ------- | ------- | ------- | ------- |

*The parentheses contain the corresponding theoretical *t* and F values at (*P*=0.05).


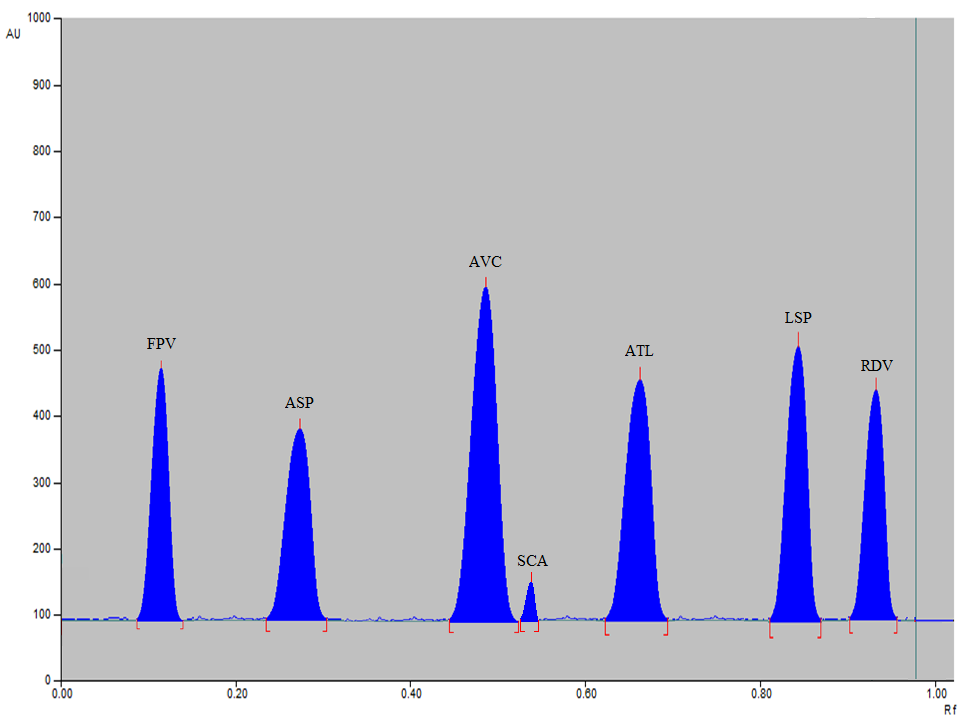


**Figure S1:** HPTLC-densitogram of the six drugs in the presence of salicylic acid (SCA) under the specified chromatographic conditions.

**Figure S2:** Normal plots of residuals for the six drugs.


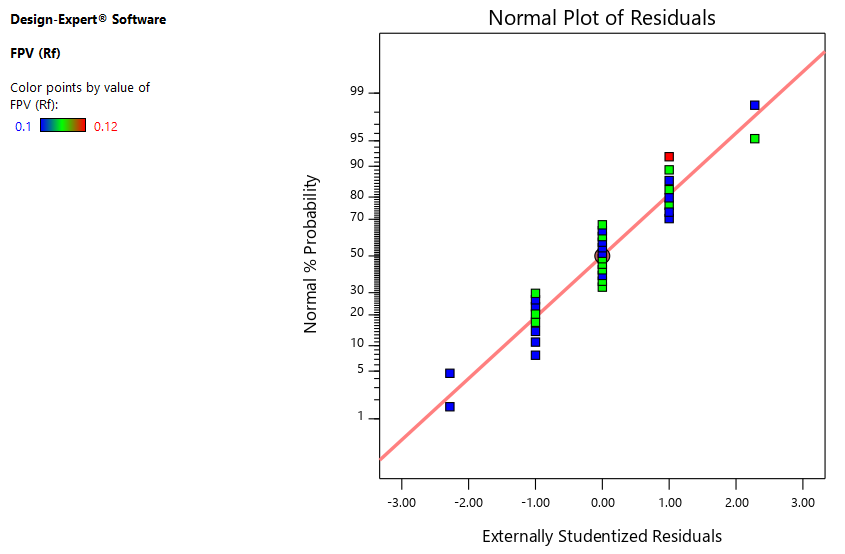

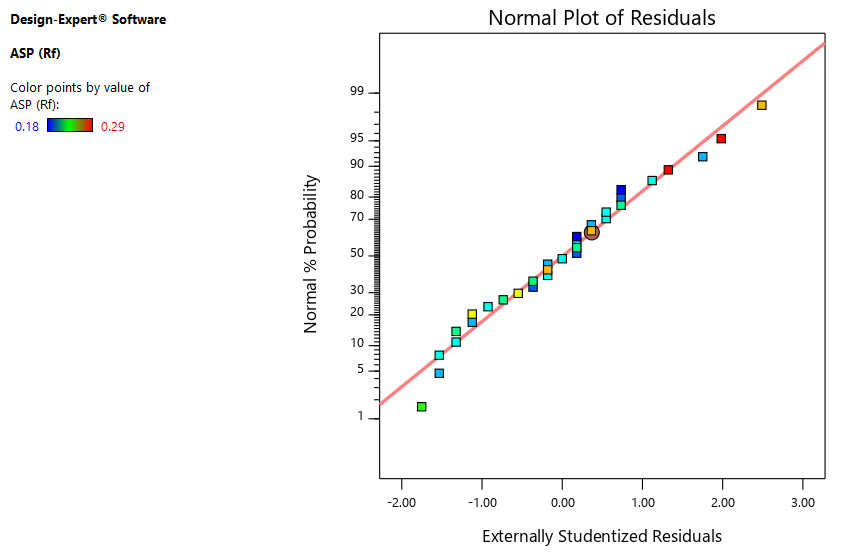

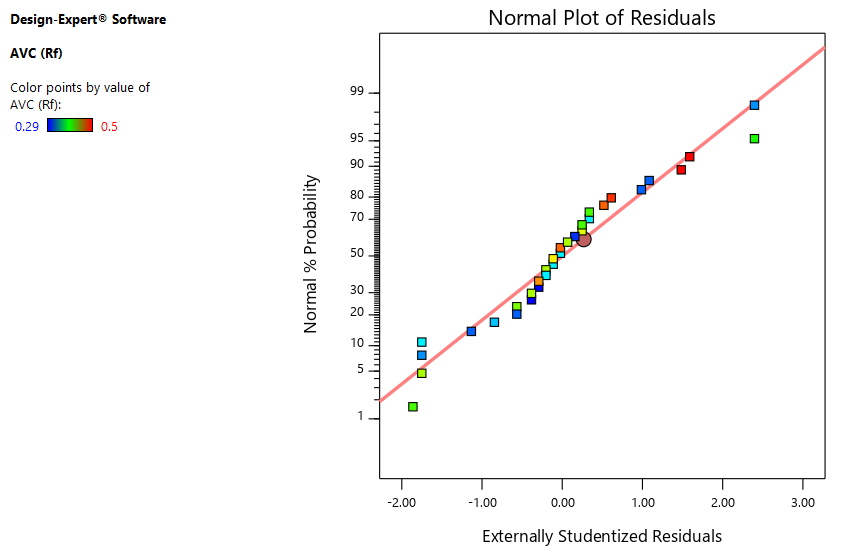

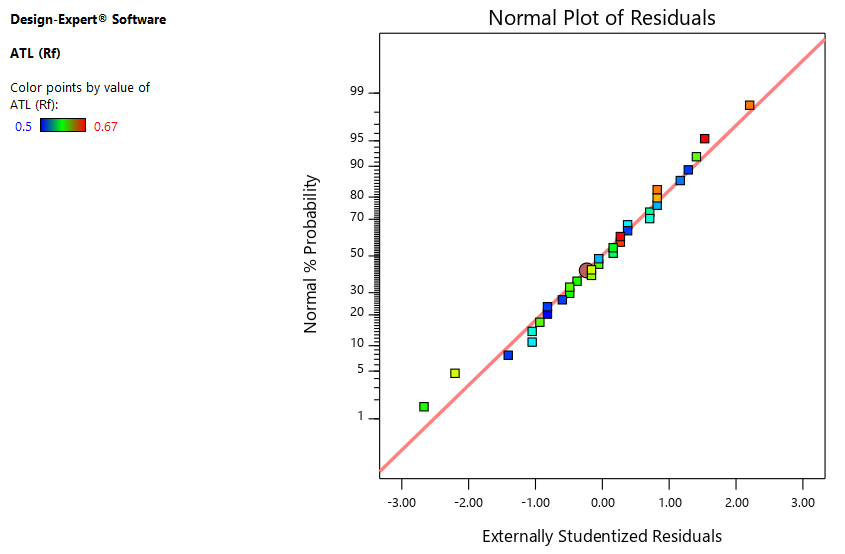

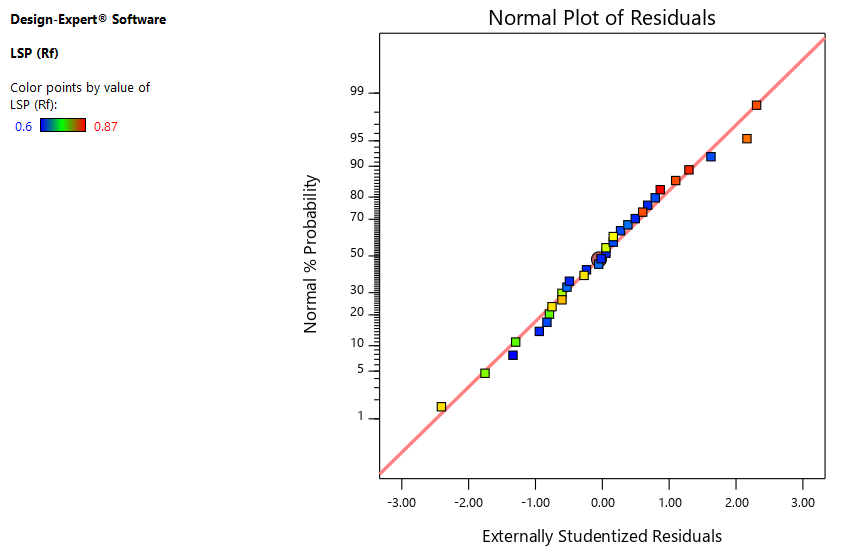

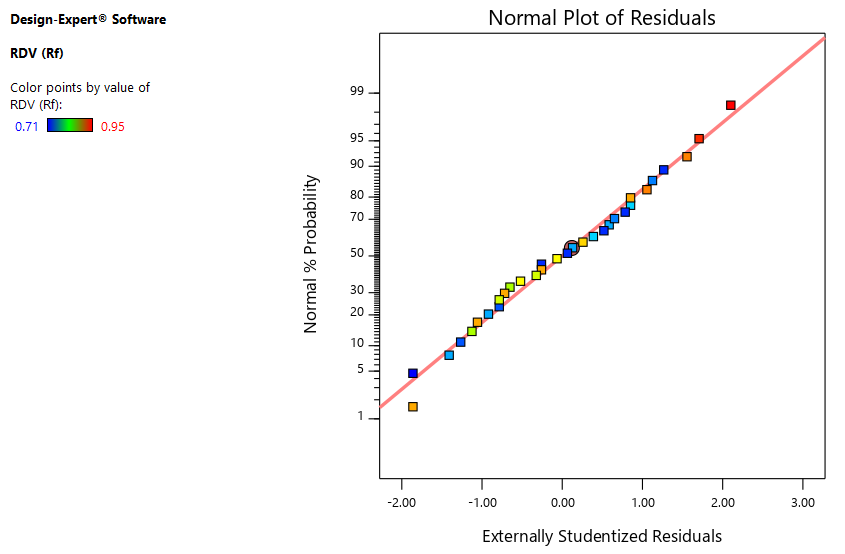


**Figure S3:** Plots showing residuals versus predicted R_f_ values for the six drugs.


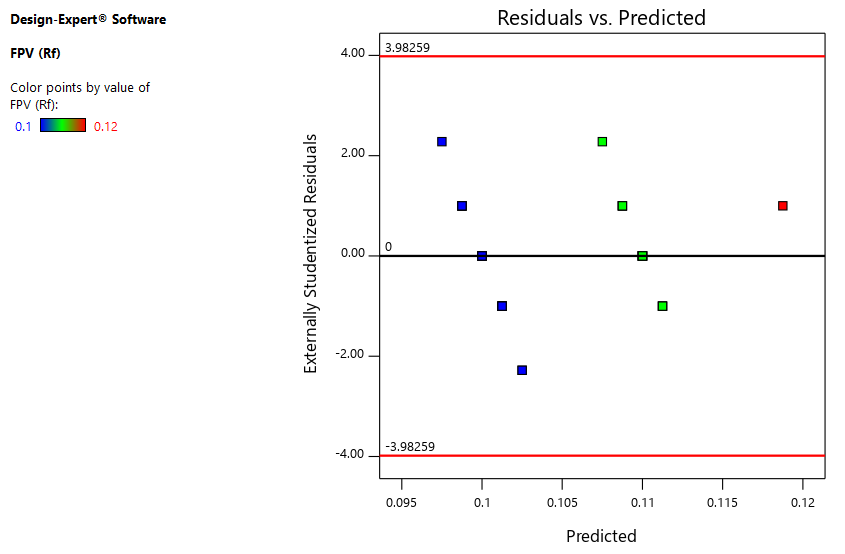

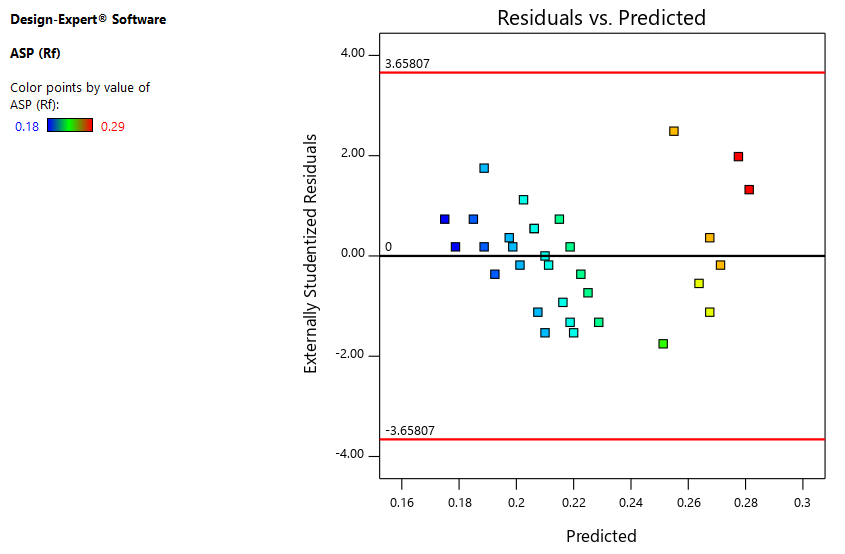

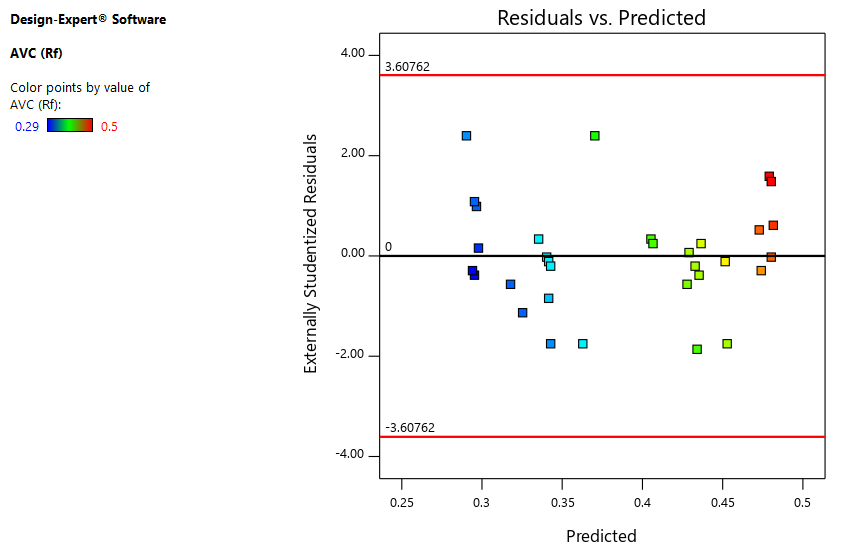

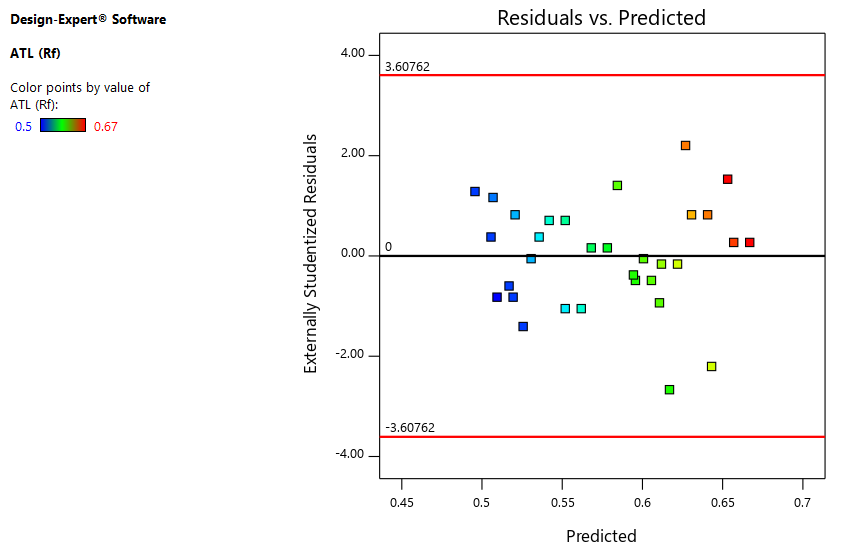

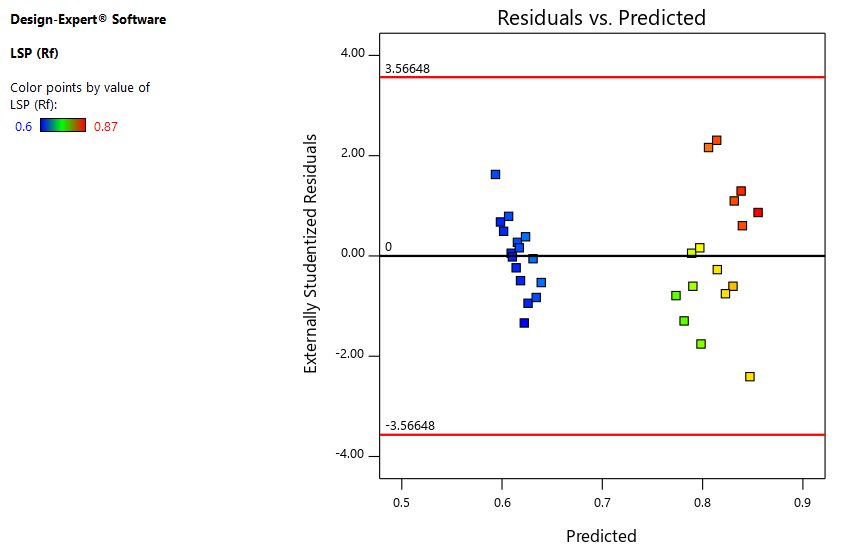

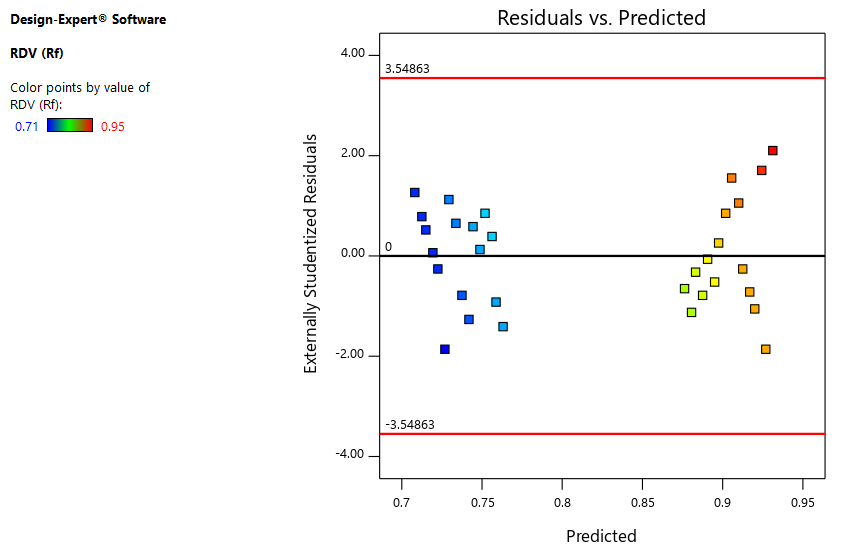


**Figure S4:** Plots showing residuals versus each run for the six drugs.


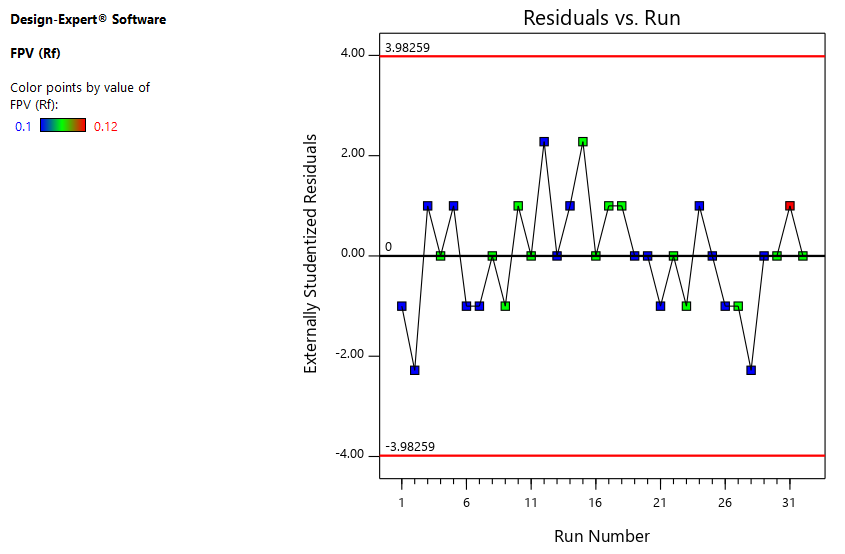

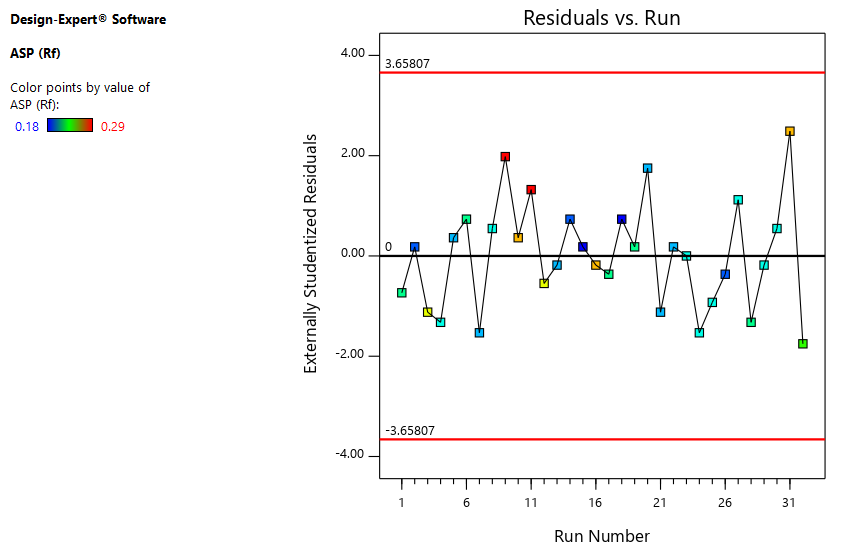

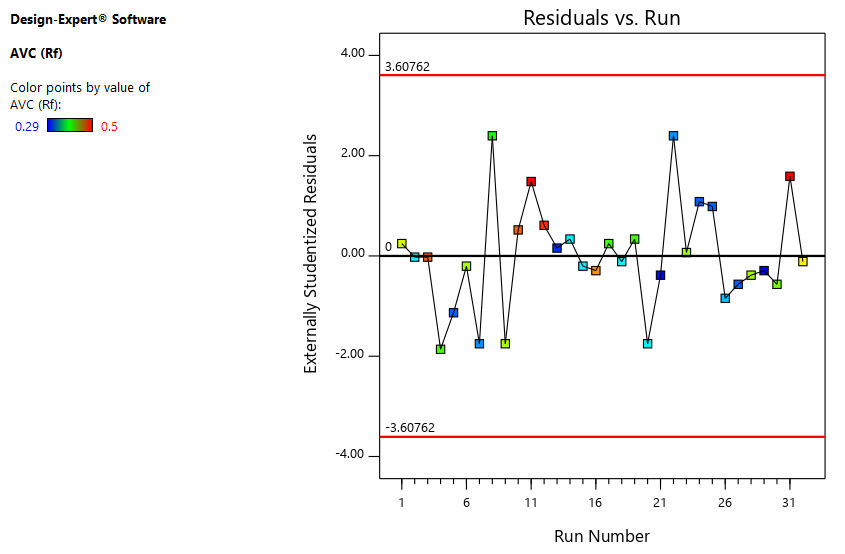

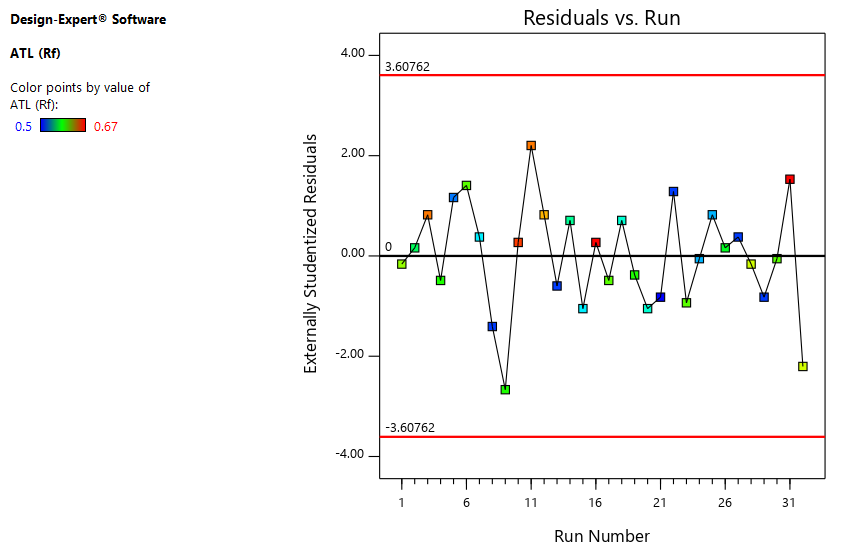

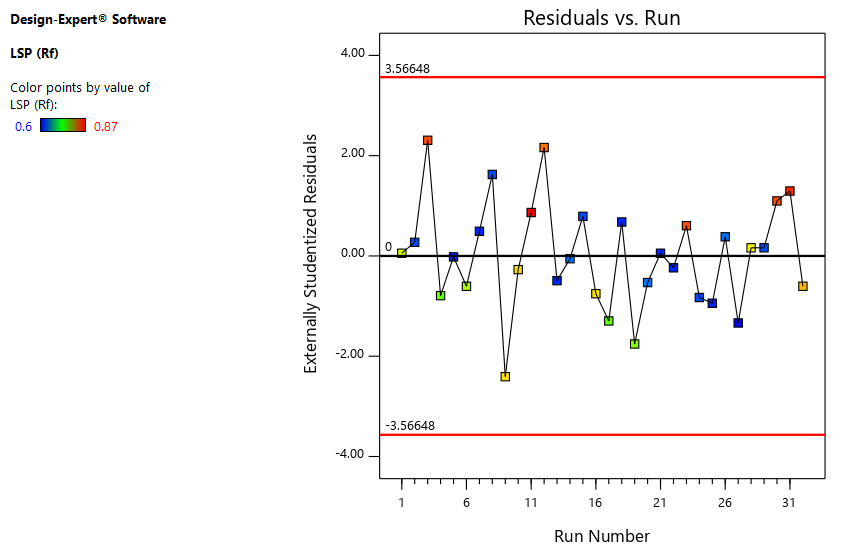

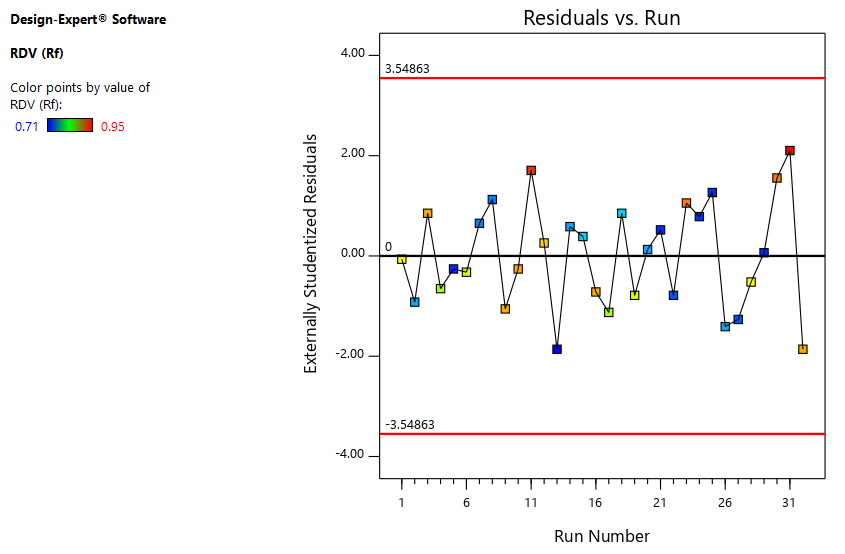


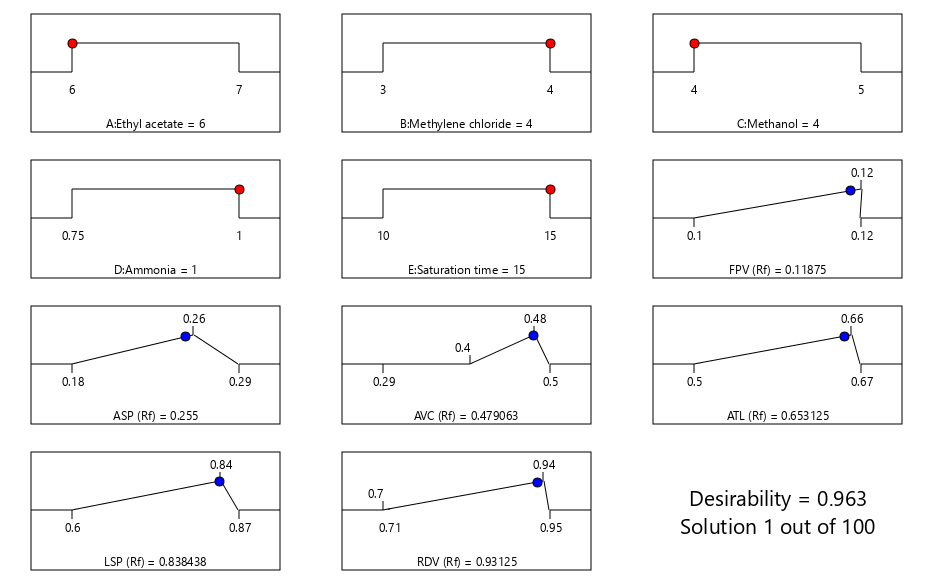


**Figure S5:** Numerical optimization ramps for the best solution (red points represent optimal variables chosen, while blue points represent predicted R_f_ values for the six drugs).


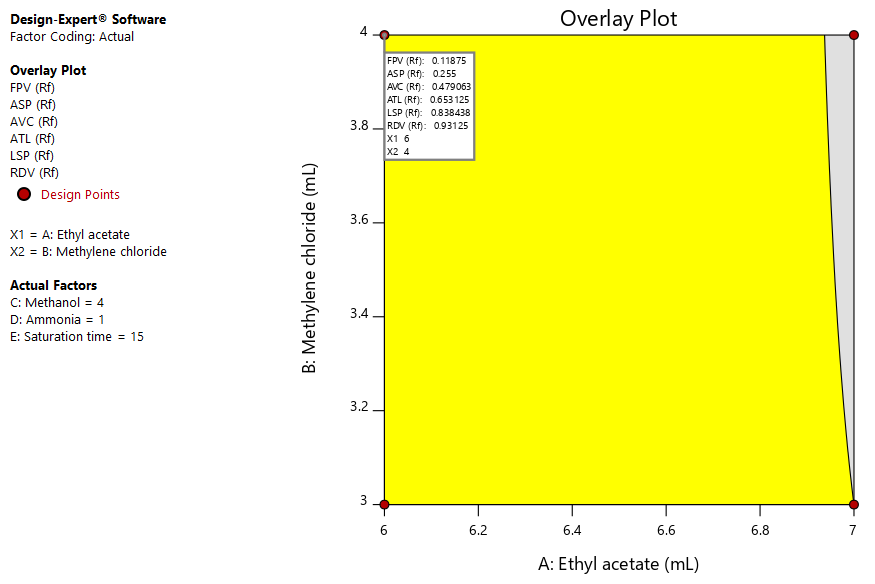


**Figure S6:** Overlay desirability plot.

**Figure S7:** 3D response surface plots for the six drugs.


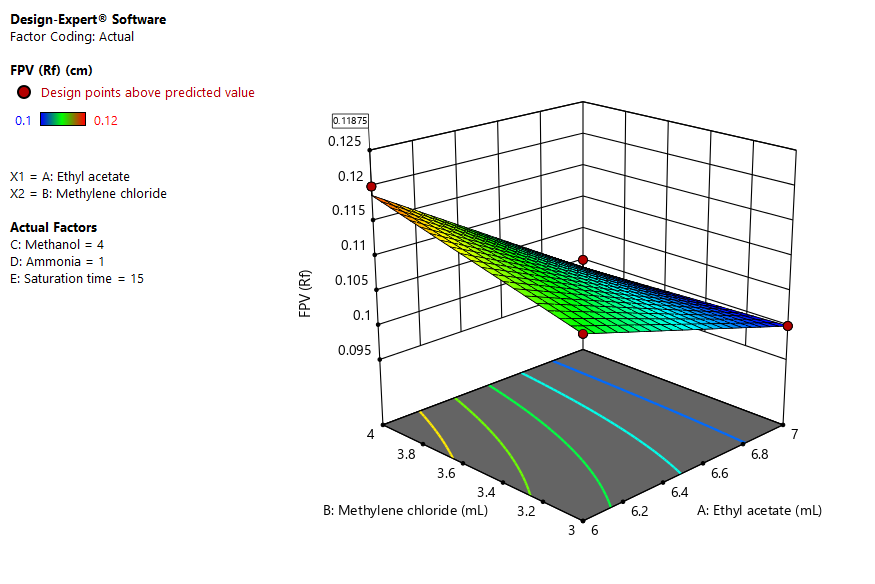

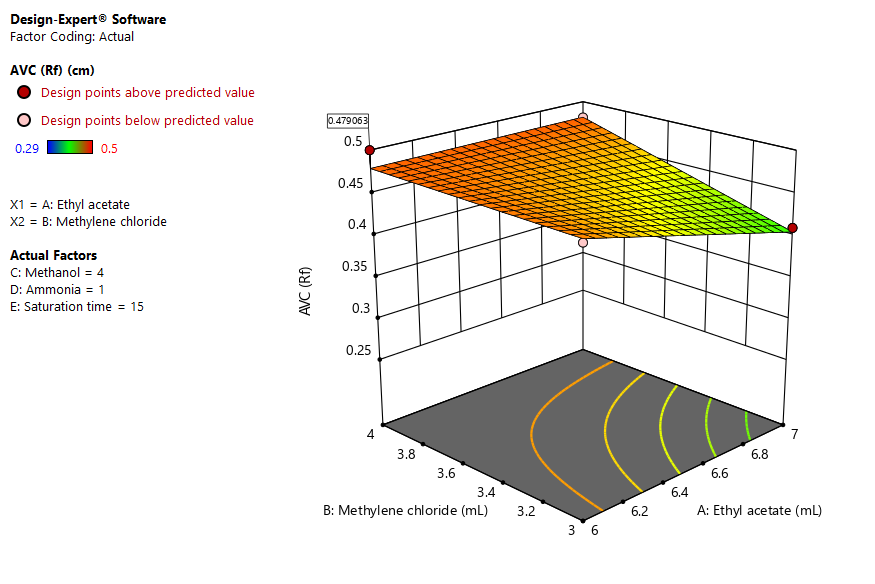

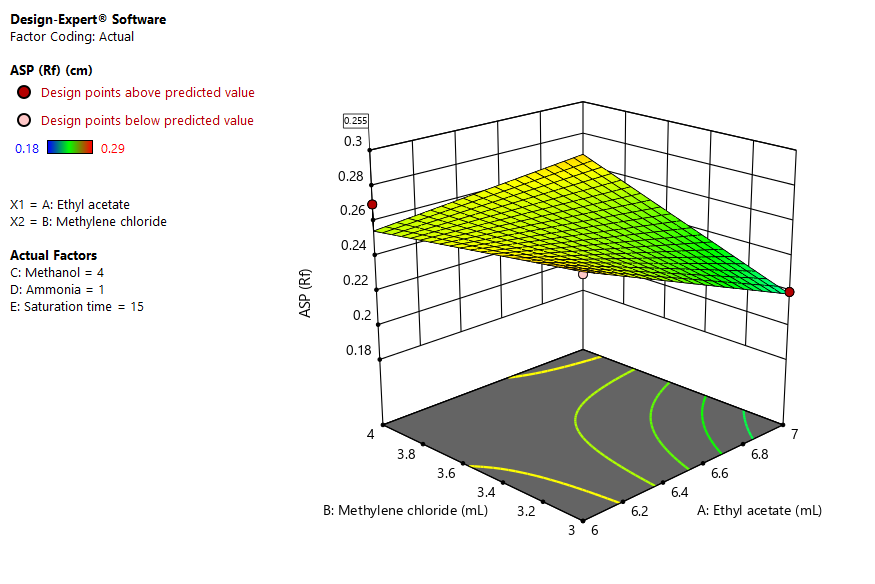

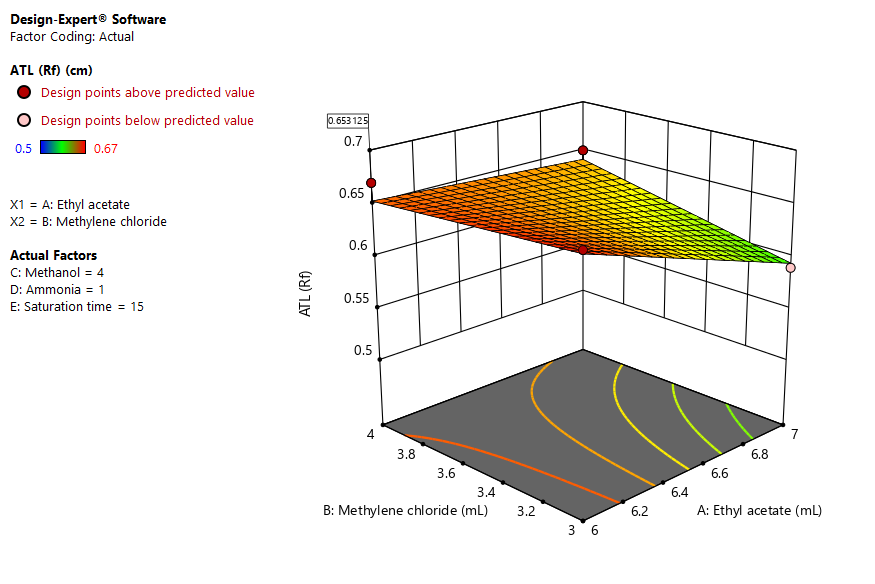

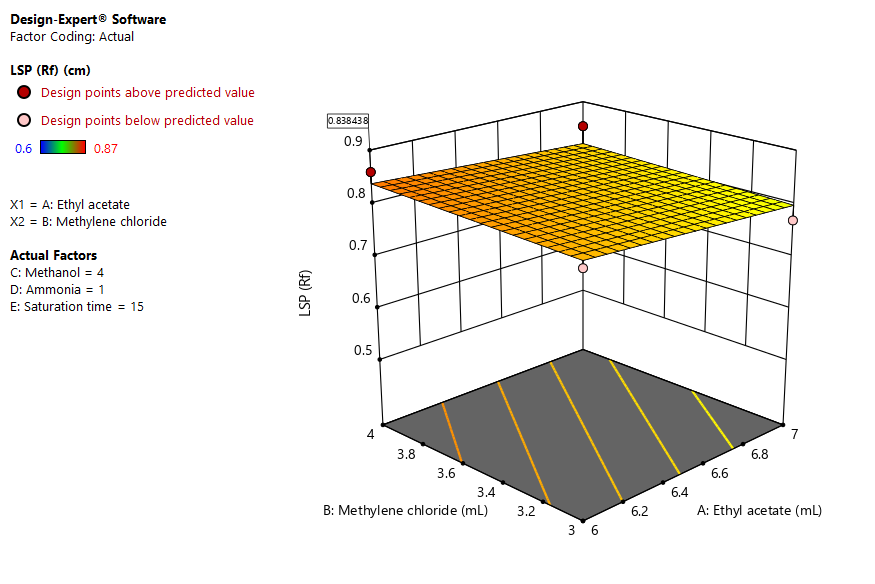

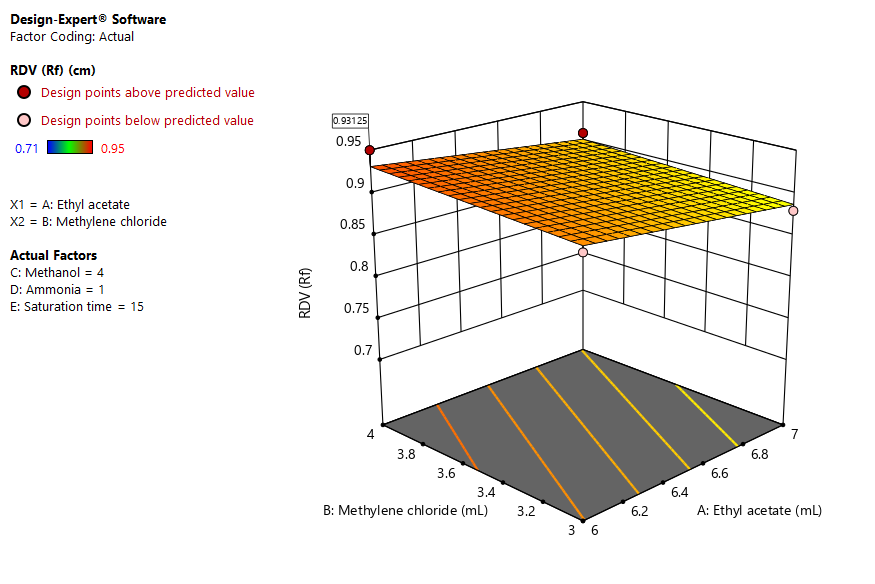


**Figure S8:** Perturbation plots for the six drugs.


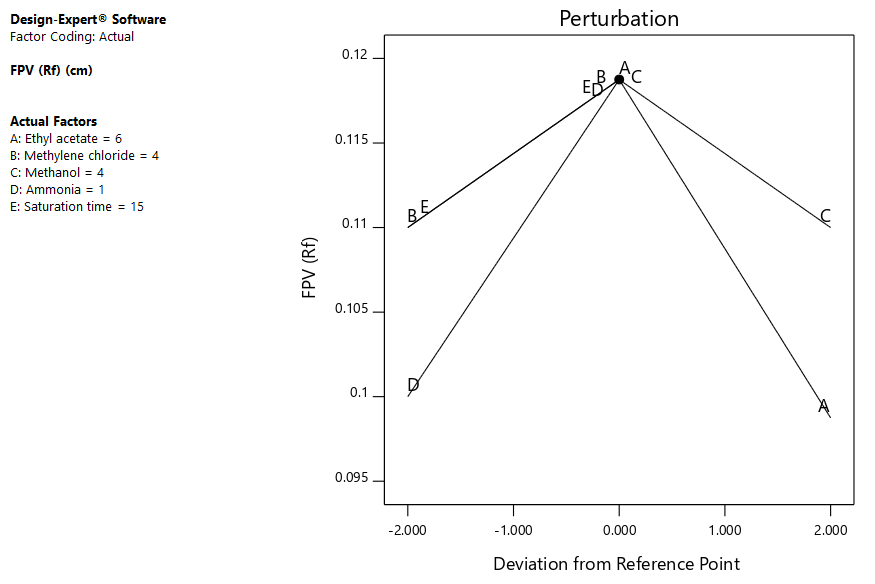

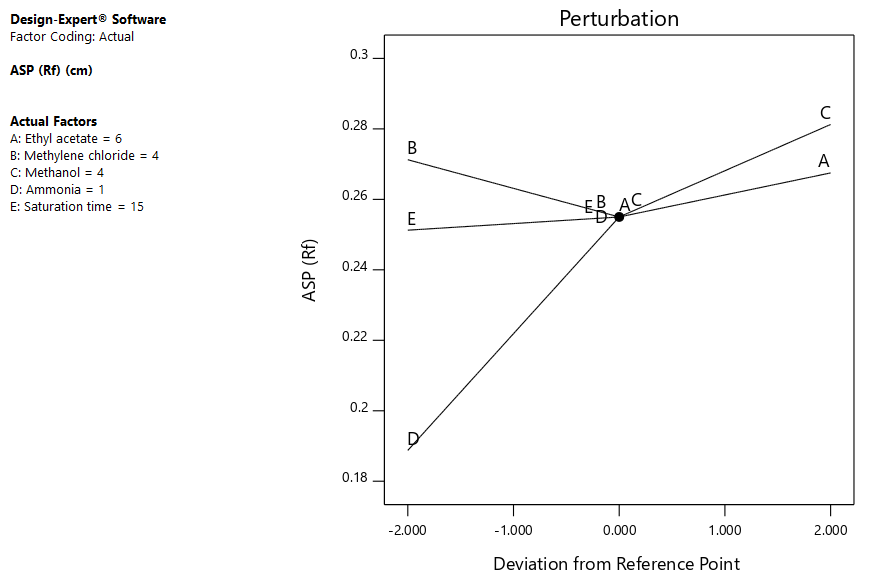

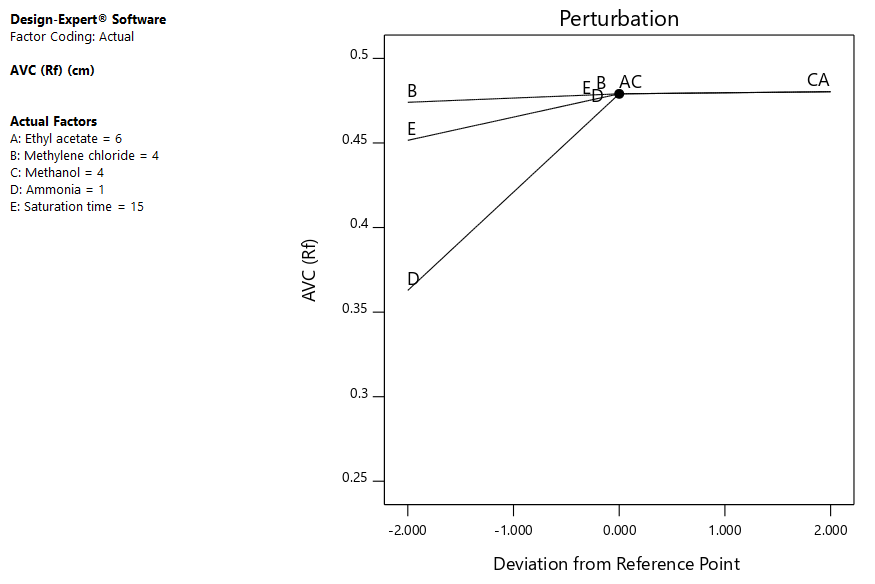

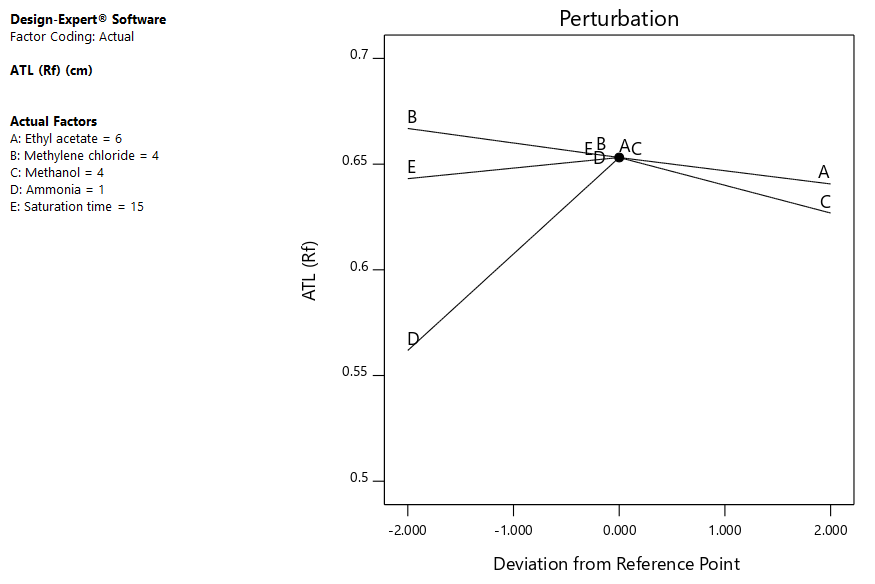

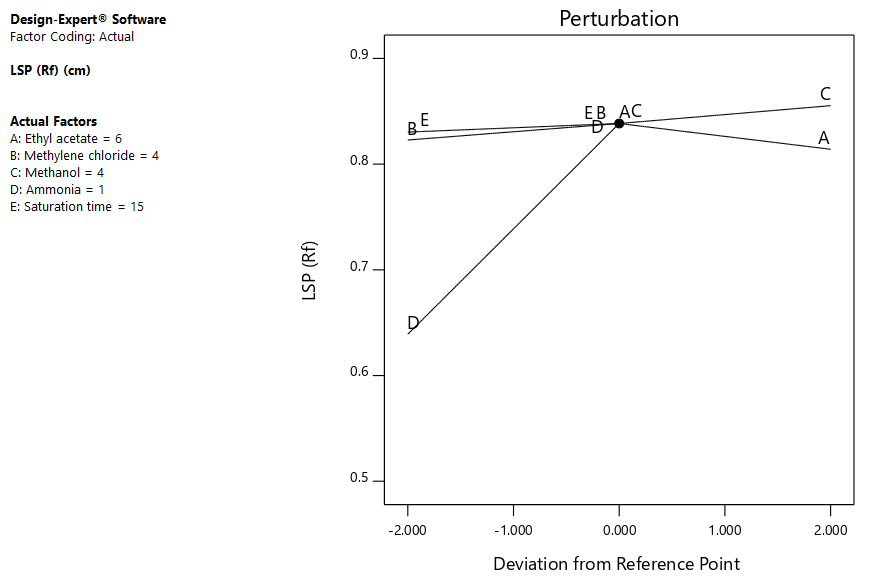

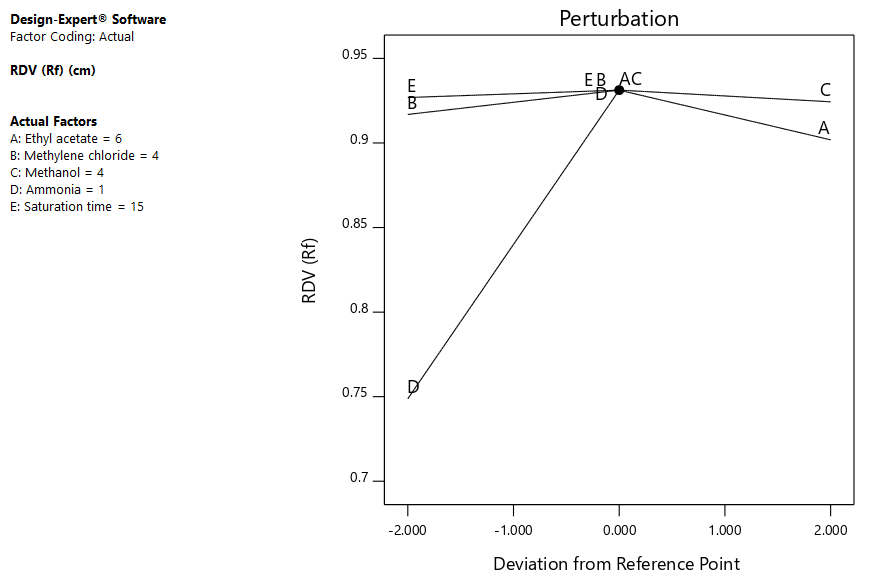


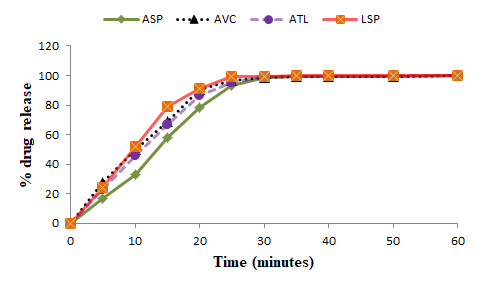


(a)


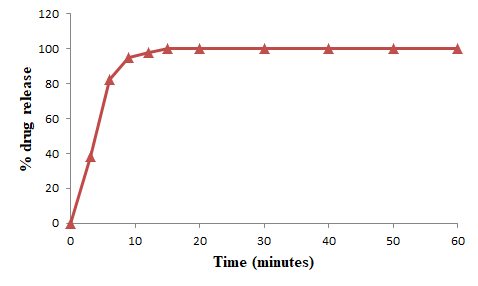


(b)

**Figure S9:** In-vitro dissolution profiles of (a) Starpill^TM^ tablets and (b) Avipiravir^®^ tablets using the suggested HPTLC approach.
